# The gut microbiome influences the bioavailability of olanzapine in rats

**DOI:** 10.1016/j.ebiom.2021.103307

**Published:** 2021-04-02

**Authors:** Sofia Cussotto, Jacinta Walsh, Anna V. Golubeva, Alexander V. Zhdanov, Conall R. Strain, Fiona Fouhy, Catherine Stanton, Timothy G. Dinan, Niall P. Hyland, Gerard Clarke, John F. Cryan, Brendan T. Griffin

**Affiliations:** a APC Microbiome Ireland, University College Cork, Cork, Ireland; b Department of Anatomy and Neuroscience, University College Cork, Cork, Ireland; c School of Pharmacy, University College Cork, Cavanagh Pharmacy Building, Cork, Ireland; d School of Biochemistry and Cell Biology, University College Cork, Cork, Ireland; e Teagasc Food Research Centre, Moorepark, Fermoy, County, Cork, Ireland; f Department of Psychiatry and Neurobehavioural Science, University College Cork, Cork, Ireland; g Department of Physiology, University College Cork, Cork, Ireland

**Keywords:** Antipsychotic, Microbiome, Pharmacokinetics, Diversity, Bioavailability, CYP, AUC_0-8h_, area under the plasma concentration *vs* time curve from time 0→ 8 hours after drug administered, AUMC_0-8h_, area under the first-moment curve from time 0→ 8 hours after drug administered, BCS, biopharmaceutical classification system, Cmax, maximum plasma concentration, CYP, Cytochrome p450 superfamily of enzymes, GF, germ-free, HPLC, High performance liquid chromatography, I.S., internal standard, LLE, liquid-liquid extraction, MDR-1, multi-drug resistance protein 1, MRT, mean residual time, OLZ, olanzapine, % CV, Percentage coefficient of variation, RISP, risperidone, RT-qPCR, reverse-transcriptase quantitative polymerase chain reaction, 5-HT, Serotonin (5-hydroxytryptamine), 5-HT-GLU, Serotonin β-D-Glucuronide, Tmax, time taken to reach maximum plasma concentration, UGT, Uridine 5′-diphospho-glucuronosyltransferase

## Abstract

**Background:**

The role of the gut microbiome in the biotransformation of drugs has recently come under scrutiny. It remains unclear whether the gut microbiome directly influences the extent of drug absorbed after oral administration and thus potentially alters clinical pharmacokinetics.

**Methods:**

In this study, we evaluated whether changes in the gut microbiota of male Sprague Dawley rats, as a result of either antibiotic or probiotic administration, influenced the oral bioavailability of two commonly prescribed antipsychotics, olanzapine and risperidone.

**Findings:**

The bioavailability of olanzapine, was significantly increased (1.8-fold) in rats that had undergone antibiotic-induced depletion of gut microbiota, whereas the bioavailability of risperidone was unchanged. There was no direct effect of microbiota depletion on the expression of major CYP450 enzymes involved in the metabolism of either drug. However, the expression of *UGT1A3* in the duodenum was significantly downregulated. The reduction in faecal enzymatic activity, observed during and after antibiotic administration, did not alter the *ex vivo* metabolism of olanzapine or risperidone. The relative abundance of *Alistipes* significantly correlated with the AUC of olanzapine but not risperidone.

**Interpretation:**

*Alistipes* may play a role in the observed alterations in olanzapine pharmacokinetics. The gut microbiome might be an important variable determining the systemic bioavailability of orally administered olanzapine. Additional research exploring the potential implication of the gut microbiota on the clinical pharmacokinetics of olanzapine in humans is warranted.

**Funding:**

This research is supported by APC Microbiome Ireland, a research centre funded by Science Foundation Ireland (SFI), through the Irish Government's National Development Plan (grant no. 12/RC/2273 P2) and by Nature Research-Yakult (The Global Grants for Gut Health; Ref No. 626891).

**Research in context**

**Evidence before this study**

Despite the growing evidence of a relationship between drugs and the gut microbiome, pharmacokinetic studies illustrating the potential clinical significance of such findings are still lacking. Yoo and colleagues have previously shown antibiotic-induced microbiota depletion can alter the absorption or metabolism of lovastatin, aspirin, and amlodipine and also that supplementation with the probiotic strain *Lactobacillus reuteri* reduced the bioavailability of acetaminophen possibly linked to the modulation of the metabolic activity of the gut microbiota.

**Added value of this study**

The study herein investigates if perturbations to the gut microbiota, induced by an antibiotic or probiotic mix, can translate to altered pharmacokinetics of olanzapine or risperidone in rats. This is the first pharmacokinetic study, to our knowledge, to explore microbiota-mediated metabolism of CNS-active drugs. Given that antibiotic-induced microbiota depletion precipitated a 1.8-fold increase in olanzapine bioavailability, several different mechanisms governing gut-microbiota-drug interactions were investigated. Interestingly, the microbiota-targeted interventions did not alter the pharmacokinetics of risperidone, suggesting the impact of microbiota depletion may be drug-specific.

**Implications of all the available evidence**

The results suggest that sources of variability in the composition and function of the gut microbiota may need to be considered a potential cause of altered drug pharmacokinetics and, ultimately, patient response to certain drugs.

Alt-text: Unlabelled box

## 1 Introduction

A growing body of evidence supports the role of the gut microbiota in dictating the fate and activity of drugs [1,2]. In recent times, the ability of the gut microbiota to directly metabolise drugs, and consequently influence drug pharmacokinetics and clinical outcomes, has come under increasing scrutiny [2,3]. Classically, the influence of the gut microbiome on drug pharmacokinetics was mostly limited to bacterial metabolism of drug metabolites excreted via the bile into the intestine (for example, glucuronide conjugates) leading to prolongation of drug half-life, or as commonly referred to the enterohepatic recirculation. More recently, studies have highlighted how the microbiome may directly metabolise a variety of drugs and how the metabolic activity of the microbiome can both activate or degrade drugs [4]. The microbiome can also produce metabolites which compete with drugs for drug-metabolising enzymes or can modulate the latter's gene expression [2,5]. While the metabolic capacity of the microbiome to metabolise a diverse array of drugs is clear, what remains unclear is the extent to which this significantly impacts clinical pharmacokinetics of drugs *in vivo* (i.e.,>20% change in systemic drug concentrations) [6]. Similarly, changes in the gut microbiome have been increasingly reported as a potential source of inter-individual variability, but a limited number of studies have explored whether changes in gut microbiome can lead to clinically significant changes to *in vivo* pharmacokinetics.

Previous work from our laboratory has linked the gut microbiome to alterations in drug pharmacodynamics; antibiotic-induced microbiota depletion attenuated olanzapine (OLZ)-associated metabolic dysfunction in rats [7,8]. This is consistent with studies in germ-free (GF) mice who do not gain weight following oral administration of OLZ [9]. Although the mechanistic basis for this association remains unclear, most attention has been placed on the antimicrobial effects of OLZ [9,10]. Microbiota depletion is considered a crucial factor in the reduction of metabolic side effects associated with this drug. A similar role of the gut microbiome in risperidone (RISP)-associated metabolic side effects has also been suggested [11], and a recent meta-analysis of both animal and human studies linked antipsychotic-induced metabolic dysfunction to the gut microbiome [12]. This study aimed to extend this research further to assess whether perturbations to the gut microbiome could also alter the pharmacokinetic profile of these two antipsychotic drugs.

OLZ and RISP are both centrally active, poorly water-soluble drugs, according to pharmacopoeial specifications, with comparable half-lives, and are widely used clinically (see Table 1). Although both are associated with microbiome-mediated weight gain *in vivo* [7,11], and antimicrobial effects *in vitro* [9,10], they exhibit some differences in pharmacokinetic profiles, chemical structure and microbiota exposure. OLZ is cleared primarily via hepatic metabolism, with less than 10% of the drug excreted unchanged in the urine [13], and 30% detected in faecal material [14,15]. The cytochrome-P450 (CYP) system metabolises OLZ, principally by *CYP1A2*, and to a lesser extent by *CYP2D6*. OLZ displays a substantial first-pass effect, with ~40% of the oral dose subject to pre-systemic metabolism. OLZ is also subject to glucuronidation via phase II enzymes UDP-glucuronosyltransferase *(UGT) 1A4* and to a lesser extent, *UGT2B10*. In the case of RISP, *CYP2D6*-mediated metabolism to form 9-hydroxyrisperidone is considered the major metabolic pathway in humans; *CYP3A4* also plays a minor role in the generation of this active metabolite [16]. RISP undergoes little to no phase II hepatic metabolism, and most of the oral dose, approximately 70%, is recovered unchanged in the urine [16,17].

**Table 1:** Overview of the physiochemical properties of the antipsychotics.

Drug name, IUPAC name, and structure	Dosage	Physiochemical properties			
		Mol.wt.	Log P	pKa	Solubility (H_2_O)
Olanzapine *2-methyl-4-(4-methylpiperazin-1-yl)-10H-thieno [2,3b] [1,5]benzodiazepine*	Target: 10 mg/day	312 g/mol	2	7.37^a^	0.039 mg/ml
Risperidone *3- [2- [4-(6-fluoro-1,2-benzoxazol-3-yl) piperidin-1-yl]ethyl]-2-methyl-6,7,8,9-tetrahydro-pyrido [1,2-a]pyrimidin-4-one*	Range: 2–10 mg/day	410 g/mol	3.49	8.24^a^	0.064 mg/ml^b^

Log P: partition co-efficient;

a [18];

b [19]

The primary objective of this study was to assess whether perturbations to the gut microbiota would influence the oral bioavailability of either OLZ or RISP. Two models of microbiota-targeted interventions in rats were explored: one rat group received an antibiotic cocktail over two weeks to deplete the microbiota, and a second group received a commercially available cocktail of probiotics (VSL#3) over the same period. The impact of these microbiota-directed interventions on the oral bioavailability of both drugs was subsequently compared to control rats.

## 2 Methods

### 2.1 Animals

Adult male Sprague Dawley rats (*n* = 6–7/group; 200–250 g on arrival) were obtained from Envigo UK. Based on our experience in employing these microbiota-targeted intervention animal models coupled with results from published comparative bioavailability studies, the following statistical parameters were employed in powering this study design: the power (1-β) of the study set as 80%, calculations based on an effect size of 1.33 (estimated from previous studies with similar experimental design), with the significance level (α) set as 5%. These calculations were performed on G*Power (version 3.1.9.2 University Kiel, Germany). Under these conditions, a total sample size of 6–7 was determined necessary to achieve significance for comparisons. They were housed 3–4 per standard cage in a conventional animal facility and maintained under a 12 h light/dark cycle, provided with chow and water *ad libitum*. Animals were randomly assigned to different cohorts using a random number generator. Rats in the same cage underwent the same treatment to avoid confounding factors such as a coprophagic effect. Animals were acclimated to housing conditions for one week before the experimental treatment.

### 2.2 Ethics

Animal experiments comply with the ARRIVE guidelines and were carried out in accordance with the U.K. Animals (Scientific Procedures) Act, 1986, and associated guideline European Directive 2010/63/EU. Approval by the Animal Experimentation Ethics Committee of University College Cork (AE19130/P049) was obtained before the commencement of all animal-related experiments. The body weight of each rat was regularly monitored every three days to assess weight-loss induced by probiotic or antibiotic treatment.

### 2.3 Antibiotic and probiotic treatment

**Fig. 1:**
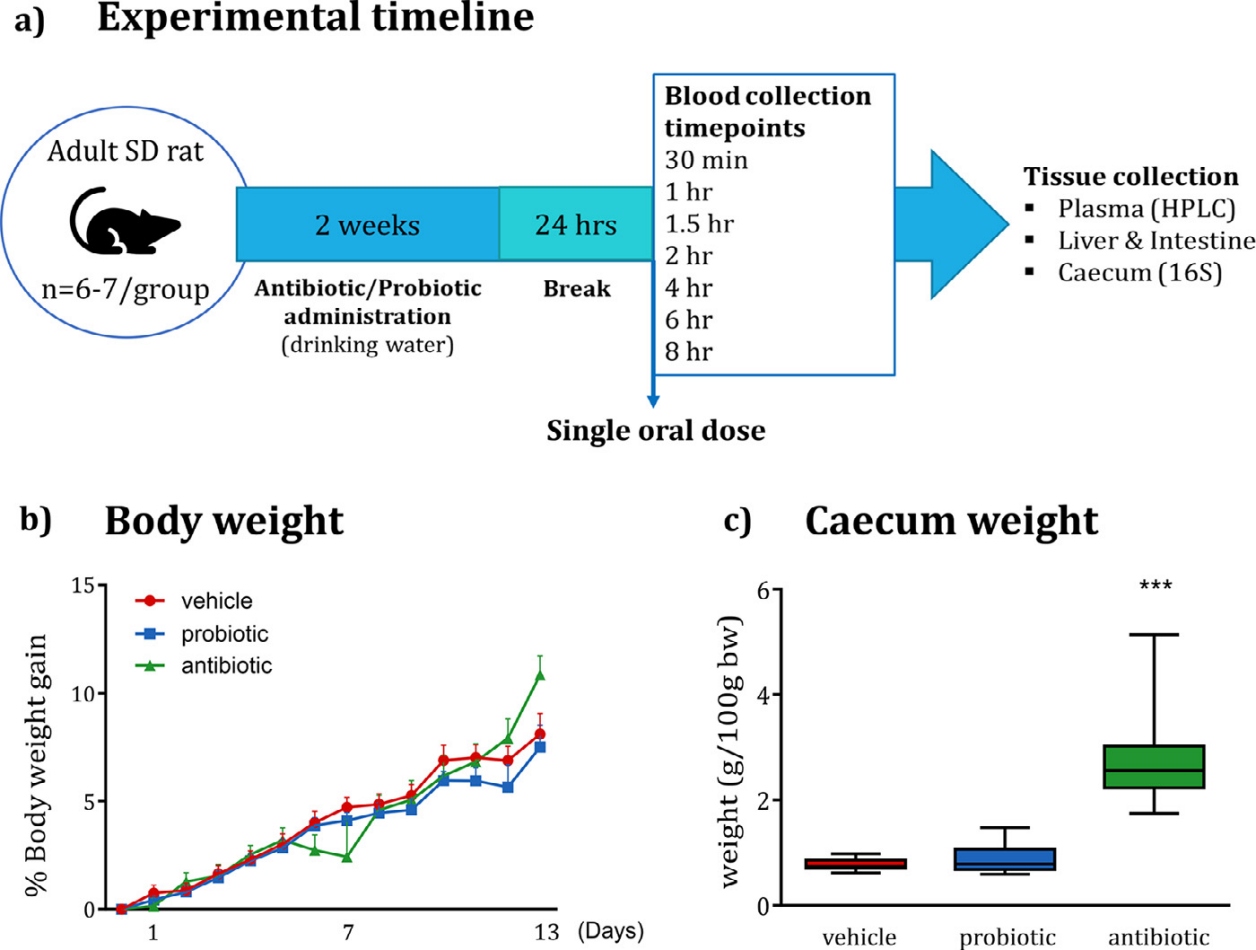
**Experimental timeline, body weight, and caecum weight. (a)** Experimental timeline. **(b)** Neither probiotic nor antibiotic administration influence the body weight gain. **(c)** Antibiotic administration increases caecum weight (KW *p* = 0.000, U = 0, *p* = 0.000). Caecum weight is expressed as median + min-to-max values. *p < 0.05 (*n* = 13–14/group).

Antibiotics or probiotics were administered for 14 days in the drinking water. The antibiotic cocktail consisted of ampicillin 1 g/L, vancomycin 500 mg/L, and imipenem 250 mg/L [adapted from [20]], and the solution was freshly prepared every second day. The probiotics consisted of VSL#3, a commercially available multi-strain preparation, which was administered in a dose of 5•10^10^ bacteria/kg/day [21]. VSL#3 contains four strains of lactobacilli *(Lactobacillus paracasei, L. plantarum, L. acidophilus and L. delbrueckii subsp. bulgaricus)*, three strains of bifidobacteria *(Bifidobacterium longum, B. breve and B. infantis)* and *Streptococcus salivarius subsp. Thermophiles.* The probiotic solution was prepared daily just before the start of the dark cycle (Fig. 1*a*).

### 2.4 Antipsychotic administration and sample collection

The two-week pre-treatment with either vehicle, probiotics or antibiotics, was followed with a 24 h intervention-free period to limit any drug-antibiotic or -probiotic interactions. After the 24 h period elapsed, a single dose of OLZ (20 mg/kg) or RISP (15 mg/kg), suspended in 0.1% v/v methylcellulose, was administered to the rats via oral gavage. Following administration of the antipsychotics, whole blood samples were collected into heparinized tubes from the tip of the tail at different timepoints (30 min, 1 h, 1.5 h, 2 h, 4 h, 6 h, 8 h). The plasma was harvested by centrifugation at 3000 g for 10 min and stored at −20 °C until further analysis. Tissue samples from the liver (frontal lobe), caecum, duodenum, and colon were also taken at dissection and immediately snap-frozen. At dissection, caecal content was also isolated from the caecum and stored separately. Faecal samples were collected periodically during the pre-treatment period. All samples were kept at −80 °C until further analysis.

### 2.5 High performance liquid chromatography (HPLC) detection of the drugs in plasma and caecal contents

The liquid-liquid extraction (LLE) protocol and HPLC conditions employed for the detection of OLZ and RISP in plasma samples was based on previously published methods with some modifications [22,23]. Detailed information on plasma and caecum preparation, as well as drug extraction, is presented in the *Supplemental Methods*. The accuracy of the technique was determined by carrying out the extraction procedure on plasma or caecal content samples spiked with known concentrations of the drug of interest, followed by HPLC analysis. For analyte identification and quantification, calibration standards were prepared by spiking 10 or 20 µl of working standard solutions (RISP or OLZ) into 90 µl of blank rat plasma or 200 µl of blank rat caecal contents respectively at final concentrations of 15.6–2000 ng/ml. Calibration curves were generated by plotting the [peak area of analyte versus internal standard (I.S.); OLZ], or analyte peak area (RISP), versus the concentration of the analyte using least-square linear regression. The correlation coefficients of the calibration curves were greater than 0.94. The percentage coefficient of variation (% CV) for the HPLC method, assessed over five days, was less than 16% for both drugs. Further information on the HPLC equipment is detailed in the *Supplemental Methods.*

### 2.6 Pharmacokinetic analysis

The maximum plasma concentration (Cmax) and the time taken to reach Cmax (Tmax) for OLZ and RISP were estimated directly from the plasma concentration-time profiles. Pharmacokinetic parameters, including the area under the plasma drug curve (AUC), clearance (Cl), and mean residual time (MRT), were all calculated using a non-compartmental model. The AUC was calculated using the linear trapezoidal method from the first to the last measured plasma concentration, i.e., AUC_0-8h_. Antipsychotic drug clearance from plasma was estimated by dividing AUC by the administered drug dose, and MRT was calculated by dividing AUC_0-8h_ by the area under the first-moment curve (AUMC_0-8h_). Each point in the PK curve represents the average of seven independent samples (*n* = 7/experimental group/drug), apart from the OLZ 1h time-point only where plasma samples were harvested from 3 rats (*n* = 3). The reduced numbers at this specific time-point arose due to difficulties in obtaining free-flowing blood from the tail of certain rats. As a safety and welfare precaution, we decided to not apply any undue pressure to the tail to minimise the amount of stress inflicted to these animals and jeopardise the collection of later time-points. The OLZ or RISP concentration in each individual plasma sample was calculated based on the average of duplicate readings.

### 2.7 Microbiota composition of the caecal content

DNA was extracted from the caecal content using the Qiagen QIAmp Fast DNA Stool Mini Kit coupled with an initial bead-beating step. The V3-V4 hypervariable region of the 16S rRNA gene was amplified and prepared for sequencing as outlined in the Illumina 16S Metagenomic Sequencing Library protocol. Samples were sequenced at the Teagasc Sequencing Facility (TFRC, Moorepark) on the Illumina MiSeq platform using a 2 × 250 bp kit. Reads were assembled, processed and analysed following the pipeline described in *Supplemental Methods*.

### 2.8 RNA extractions, reverse transcription, and quantitative RT-qPCR

Total RNA was extracted from the intestinal tissue and the front lobe of the liver with the mirVana^TM^ miRNA isolation kit (Thermo Fisher Scientific/Ambion) following the manufacturer's protocol. A Nanodrop 1000 (Thermo Scientific, UK) was used to determine RNA concentration. RNA was reverse transcribed using a high capacity cDNA reverse transcription kit (Thermo Fisher Scientific/Applied Biosystems) in a G-storm thermocycler (G-storm, Surrey, UK). Genes of interest (listed in the *Supplemental Methods*) were amplified using SYBR Green primers. Each transcript value was calculated as the average of triplicate samples across experimental conditions. Values were normalized to β-actin or GAPDH. Data were analysed with the comparative cycle threshold method (2^−ΔΔCt^) [24], and presented as a fold change *vs* vehicle group.

### 2.9 Protein extractions and Western blots

Total proteins from liver samples were extracted through sonication, BCA quantification (Thermo Fisher Scientific), and heat denaturation. Protein levels were detected on SDS-page gels using appropriate primary antibody dilutions against *CYP1A2* (1:5000; Abcam ab22717), *CYP2D1* (1:1000; Enzo BML-CR3210), *CYP3A1* (1:1000; Millipore AB1253), UGT1A (1:500; Santa Cruz sc-271268), secondary antibody HRP-conjugated to rabbit or mouse (1:10000; Thermo Fisher Scientific). For CYP1A2, CYP2D1 and CYP3A1, the control for protein loading consisted of β-actin (1:1000; Santa Cruz, sc-47778). For UGT1A the control for protein loading consisted of ERK1/2 (1:1000; Cell Signaling #9102). Quantification of protein bands was performed using ImageJ software, and protein levels were normalized to the loading control and presented as fold change relative to the vehicle group. Significance was determined by a one-way ANOVA followed by Dunnett's test.

### 2.10 Fecalase: preparation and enzymatic assay

Fecalase, the enzyme fraction of faeces [25], was prepared from approximately 70 mg fresh-frozen rat faeces collected on day 12 of the 14-day pre-treatment period, according to a modified method previously described [26]. Briefly, the faecal pellet was suspended in 1 mL potassium phosphate buffer (0.01M, pH7.4) and homogenised using a mini Bead-Beater machine for 1.5 min. The faecal suspension was centrifuged at 2000 rpm for 5 min, and the resulting supernatant was centrifuged at 10,000 rpm for 20 min. The supernatant isolated after the second centrifugation step (fecalase) was then used for the assay of enzyme activity.

For quantification of enzymatic activity, the reaction mixture, containing 50 µl fecalase, 100 µl potassium phosphate buffer (0.01M, pH 7.4) and 100 µl 4-nitrophenyl-β-D-glucopyranoside (1 mM, Sigma Aldrich) for β-glucosidase or 100 µl 4-nitrophenyl-β-D-glucuronide (1 mM, Sigma Aldrich) for β-glucuronidase, was incubated at 37 °C for 15 min. After incubation, 250 µl NaOH (0.5 N) was added to stop the reaction, and the absorbance was measured at 405 nm (UV–vis spectrophotometer). The Pierce BCA Protein Assay Kit (ThermoFisher Scientific) was used, following the kit protocol, to measure the total protein concentration in the fecalase samples. Enzyme activity was indicated as the amount required to catalyse the formation of 1 µmol of p-nitrophenol per minute under the standard assay conditions.

### 2.11 Fecalase: *ex vivo* incubation with OLZ, RISP, and Serotonin β-D-Glucuronide (positive control)

To assess whether either OLZ or RISP was subject to bacterial-derived enzymatic degradation, the drug was incubated with fecalase prepared from vehicle-, probiotic- and antibiotic-treated rats. The reaction mixture, containing 50 µl fecalase, 200 µl potassium phosphate buffer (0.01M, pH 7.4) and 50 µl OLZ or RISP (0.25 mM, in DMSO), was prepared in duplicate and incubated with agitation (450 rpm) at 37 °C for 48 h. As experimental controls, a fecalase-only reaction mix [50 µl fecalase and 250 µl potassium phosphate buffer (0.01M, pH 7.4)] and drug-only reaction mix [50 µl OLZ or RISP (0.25 mM, in DMSO) and 250 µl potassium phosphate buffer (0.01M, pH 7.4); in triplicate] were also prepared and incubated simultaneously to the experimental samples. Aliquots (60 µl) were taken at T0, T24, and T48 from each sample. Methanol was added to each aliquot (1:1) to stop the reaction. The amount of remaining OLZ or RISP was then determined using HPLC-UV. Peak areas of OLZ and RISP were normalised to the drug-only control of each corresponding day of incubation.

Previous research has illustrated the glucuronide metabolite of the monoamine neurotransmitter serotonin, serotonin β-D-Glucuronide (5-HT-GLU), is subject to deconjugation by bacterial-derived β –glucuronidase [27]. As a positive control, 5-HT-GLU was incubated using the same reaction mix as outlined above with some modifications. Each reaction mix was maintained at 37 °C for one hour (from T0 to T1), as per a previous method [27]. After an aliquot was taken at T0 and T1, the reaction was stopped with MeOH (1:1). Before HPLC-ECD analysis, an equal volume of I.S. (n-methyl 5-HT, 2 ng/20 µl in HPLC mobile phase) was added to each sample and vortex-mixed. The total volume (300 µl) was then transferred to the HPLC vial, and 20 µl of the final sample was injected onto the column for analysis. The method for the HPLC-ECD quantification of the parent compound 5-HT was based on our previously published method [28].

### 2.12 Statistics

All datasets were checked for normality (Shapiro-Wilk test) and homogeneity (Levene's test). Data that satisfied both homogeneity and normality tests were analysed using one-way ANOVA followed by Dunnett's test. The corresponding data are presented as mean + SEM and include the following datasets: body weight, AUC bar plots, duodenal and hepatic gene expression, hepatic protein levels and fecalase readout. The plasma pharmacokinetic curves and parameters (Fig. 4a–c) are presented as mean + SD to clearly illustrate subject variability. In order to explore differences in the drug concentrations over time, a repeated measures ANOVA was also performed in conjunction with Tukey's multiple comparisons test. Datasets that did not satisfy the assumptions of homogeneity and normality were analysed using the Kruskal-Wallis non-parametric test followed by Mann-Whitney test and corrected for multiple comparisons using the Benjamin-Hochberg false discovery rate (FDR) method. These datasets (caecum weight and microbiota genera) are presented as median and min-to-max values. Spearman's correlation coefficient was employed for the analysis of associations between pharmacokinetic parameters and bacterial taxa abundance. Grubbs method was employed to test for any specific outliers [29]. The threshold for statistical significance was set at *p* < 0.05.

### 2.13 Role of funding source

The funders had no role in study design, data collection, data analysis, interpretation or writing of the report.

## 3 Results

### 3.1 Microbiota-targeted interventions did not significantly alter body weight

Over the two-week administration of either antibiotic or probiotic cocktails, rat body weight was monitored daily. There were no differences observed between control (i.e., vehicle-treated rats) and probiotic- or antibiotic-treated animals (Fig. 1*b*). Upon cessation of the study and collection of organs, the caecum weight was significantly increased in antibiotic-treated rats (Fig. 1*c*).

### 3.2 Antibiotics and probiotics differentially impacted the caecal microbiota composition

**Fig. 2:**
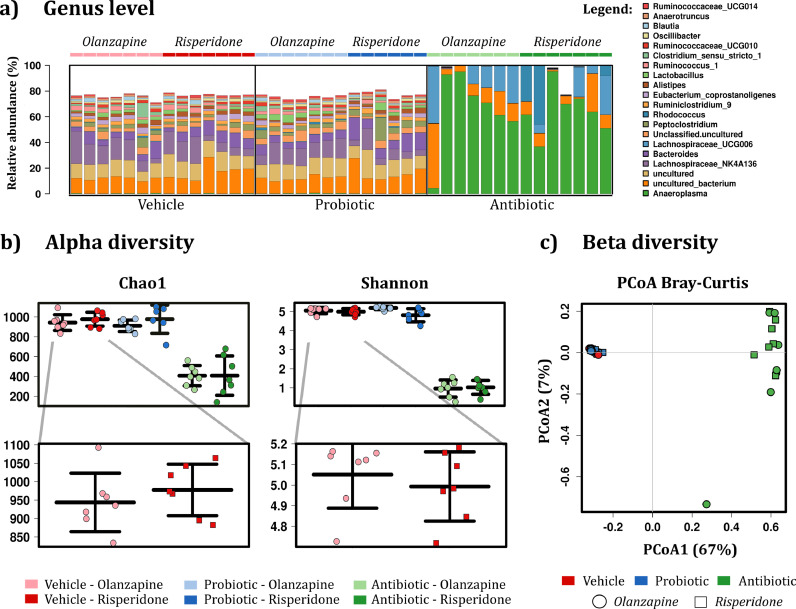
**Caecum microbiota composition (16S sequencing). (a)** Bar charts representing the taxa abundance at the genus level. The 20 most abundant taxa are shown. **(b)** Alpha diversity. Kruskal–Wallis test for Chao1 (*p* = 0.000) and Shannon (*p* = 0.000). Mann–Whitney *U* test for *Chao1* and *Shannon*: antibiotic *p* < 0.001 compared to vehicle. Data are expressed as median + min-to-max values. Samples are rarefied to read depth of 32817. **(c)** Beta diversity, principal coordinate analysis of Bray-Curtis compiled distance matrix of all microbial relative abundances compared to the vehicle group. Antibiotic animals show significant variation from the vehicle (Adonis PERMANOVA *p* < 0.001, *R*^2^ = 0.764), independent of drug treatment. (*n* = 6–7/group).

To assess the impact of the microbiota-targeted interventions, 16S sequencing of bacterial rRNA was performed on the caecum content. As expected, the sequencing revealed a significant decrease in the bacterial richness and diversity of rats treated with antibiotics as compared to the control group *(*Fig. 2*b)*. Moreover, separation by group was further illustrated through principal coordinate analysis (PCoA), with statistical support of the significant separation between the antibiotic and the vehicle group (*p* < 0.001, Fig. 2*c*). The marked difference between the antibiotic and the control group was also detected at the genus level, with several genera being depleted in the antibiotic group (Fig. 2*a*). The phylum and family microbiome signatures were also significantly disrupted by antibiotics (*Fig. S1*). In probiotic-treated rats, no marked differences were evident at the genus level, alpha, or beta diversity (Fig. 2).

Previous research from our group and others has shown that psychotropic drugs can influence the composition of the gut microbiota [30,31]. To assess whether a single dose of OLZ or RISP would impact the microbiome *per se*, we checked the alpha diversity in the vehicle group, and no significant differences were detected between OLZ- and RISP-treated rats (Fig. 2*b*).

**Fig. 3:**
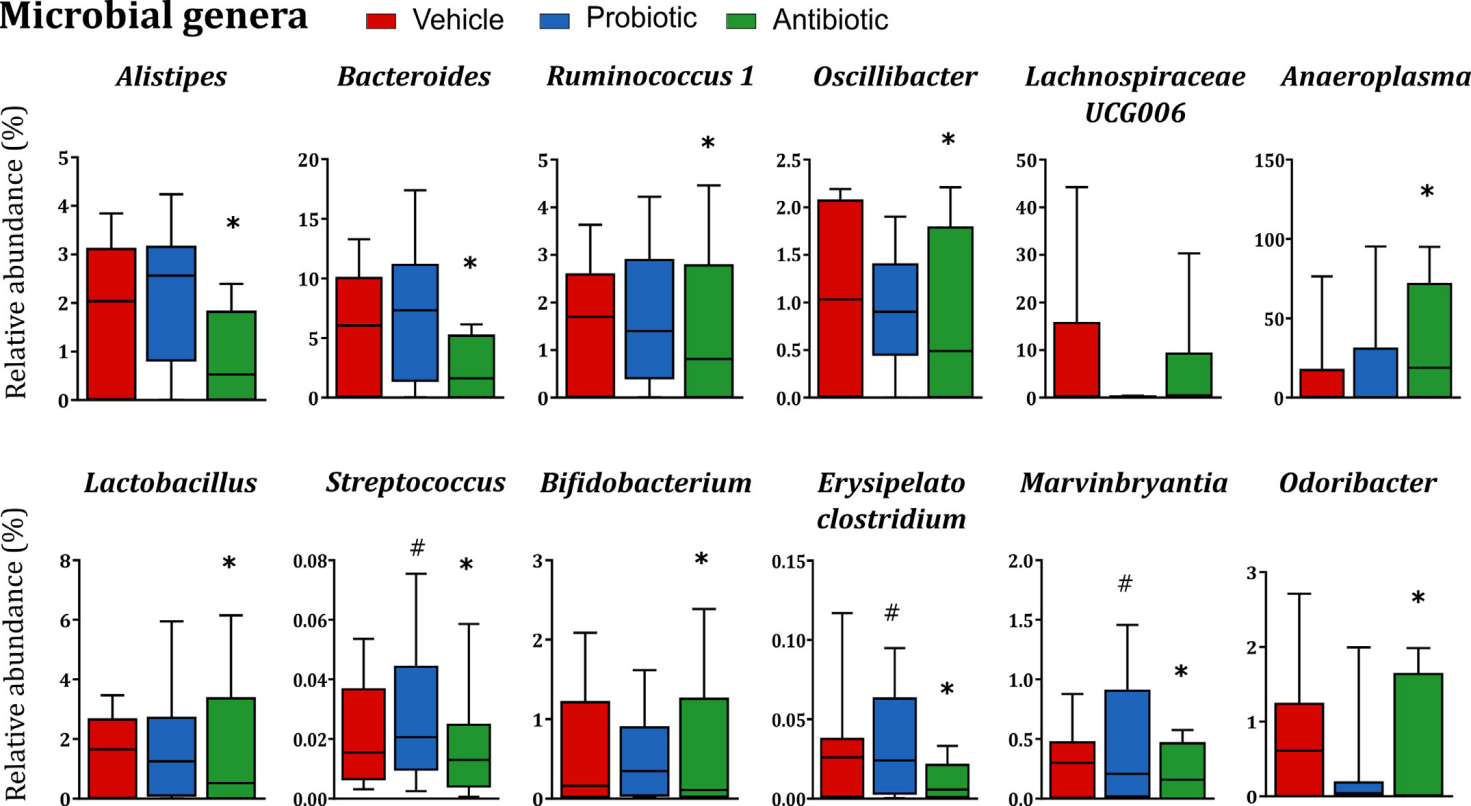
**Probiotics and antibiotics differentially affect bacterial composition at the genus level.** Many bacterial genera are completely depleted in antibiotic-treated rats. The genus *Anaeroplasma* is significantly increased in antibiotic-treated rats. *Lachnospiraceae* UCG006 is also increased but not significantly in antibiotic-treated rats. *Streptococcus, Erysipelatoclostridium, Marvinbryantia,* and *Odoribacter* are significantly increased in probiotic-treated rats compared to the vehicle group. Data are expressed as median + min-to-max values *p < 0.05 VS vehicle, ^#^*p* < 0.05 VS vehicle (*n* = 13–14/group). Data was analysed using Kruskal-Wallis non-parametric test followed by Mann-Whitney U test and corrected for multiple comparisons using the Benjamin-Hochberg false discovery rate (pFDR) method *(refer to Supplemental Material for details on statistics*)*.*

At the phylum and family level, several bacteria were altered by both microbiome-targeted interventions (*Figure S1, Tables S1, and S2*), with antibiotics causing a depletion in several families. At the genus level, antibiotics induced a broad depletion, while the genera *Anaeroplasma* was significantly increased in antibiotic-treated rats and *Lachnospiraceae* UCG006 was increased but not significantly (Fig. 3*, Table S3*). The three bacterial strains present in the VSL#3 probiotic formulation belong to the genera *Lactobacillus, Streptococcus,* and *Bifidobacterium*. Among these three taxa, only *Streptococcus* was significantly increased in the caecum of probiotic-treated rats compared to vehicle-treated rats. Other taxa that were significantly increased in probiotic-treated rats included *Erysipelatoclostridium, Marvinbryantia,* and *Odoribacter* (Fig. 3).

### 3.3 Antibiotic-induced depletion of gut microbiota influenced the oral bioavailability of OLZ

**Fig. 4:**
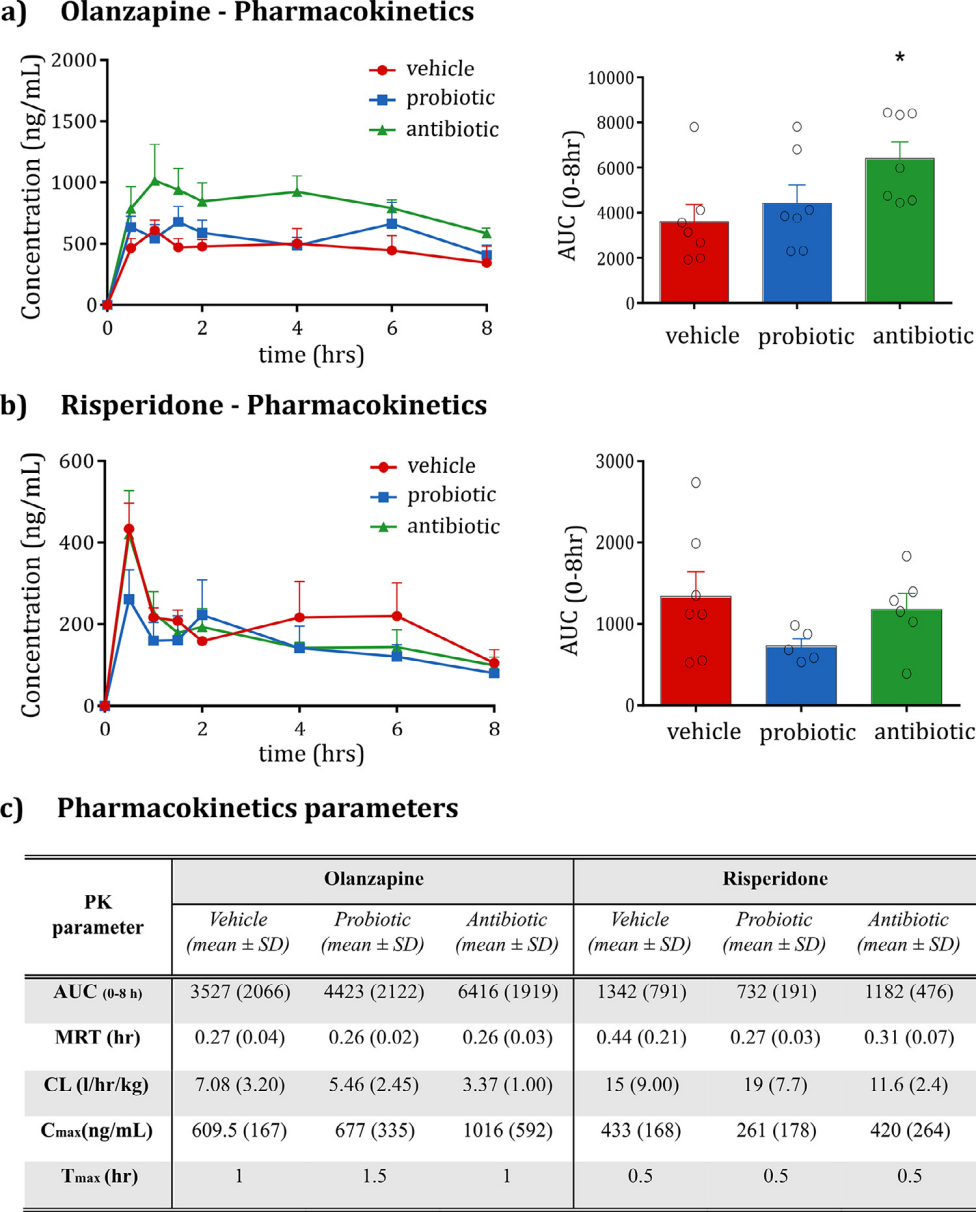
**Pharmacokinetic profile of OLZ and RISP after oral administration in rats pre-treated with the vehicle, probiotic or antibiotic. (a)** Plasma levels and area under the curve (AUC) of OLZ. Following a 24 h break, OLZ (20 mg/kg) was orally administered to rats pre-treated with vehicle, probiotic or antibiotic for 14 days (*n* = 7/group, *n* = 3 @1 h timepoint). The AUC of OLZ is increased by antibiotic administration (One-way ANOVA F_(2;20)_=3.58, *p* < 0.05; *t*-test *p* = 0.033). **(b)** Plasma levels and AUC of RISP. Following a 24 h break, RISP (15 mg/kg) was orally administered to rats pre-treated with vehicle, probiotic or antibiotic for 14 days (*n* = 6–7/group). **(c)** Pharmacokinetic parameters of OLZ and RISP after oral administration in rats pre-treated with vehicle, probiotic or antibiotic. Each point in the PK curve represents the average of 7 independent samples (*n* = 7/experimental group/drug), apart from the OLZ 1.5h time-point where plasma samples were harvested only from 3 rats (*n* = 3). The OLZ or RISP concentration in each individual plasma sample was calculated based on the average of duplicate readings. Bar plots are expressed as mean + SEM; graphs over time are expressed as mean + SD.

The plasma concentration levels of OLZ and RISP were determined after oral administration to the vehicle-, probiotic- and antibiotic-treated animals. The plasma concentration-time profile and AUC of OLZ are shown (Fig. 4*a)*. The resultant pharmacokinetic parameters are also described in Fig. 4*c*. Antibiotic-induced microbiota depletion significantly increased the AUC_0-8h_ of OLZ by approximately 82% after a single oral dose. Probiotic treatment had no impact on the pharmacokinetics of OLZ. In the case of RISP, neither of the microbiome-directed interventions significantly influenced its oral bioavailability (Fig. 4*b*).

In order to further explore differences in the profile of the drug concentrations over time, a repeated measure ANOVA was performed. OLZ plasma concentrations were significantly higher after antibiotics, relative to vehicle, at the 1.5 h and 4 h time-points (OLZ: ABX *vs* vehicle; *p* < 0.05 at both time-points). This is in line with the overall PK difference in terms of total AUC. In the case of RISP, a significant difference in plasma concentrations was found at the 6h time-point (RISP: ABX *vs* vehicle; *p* < 0.05 at 6 h). The magnitude of the difference at 6 h, however, appears to be relatively small and given the lack of a difference in total AUC, the potential impact of this effect is, thus, considered minor.

### 3.4 AUC correlated with the relative abundance of the genera *Alistipes* in OLZ- but not RISP-treated rats

**Fig. 5:**
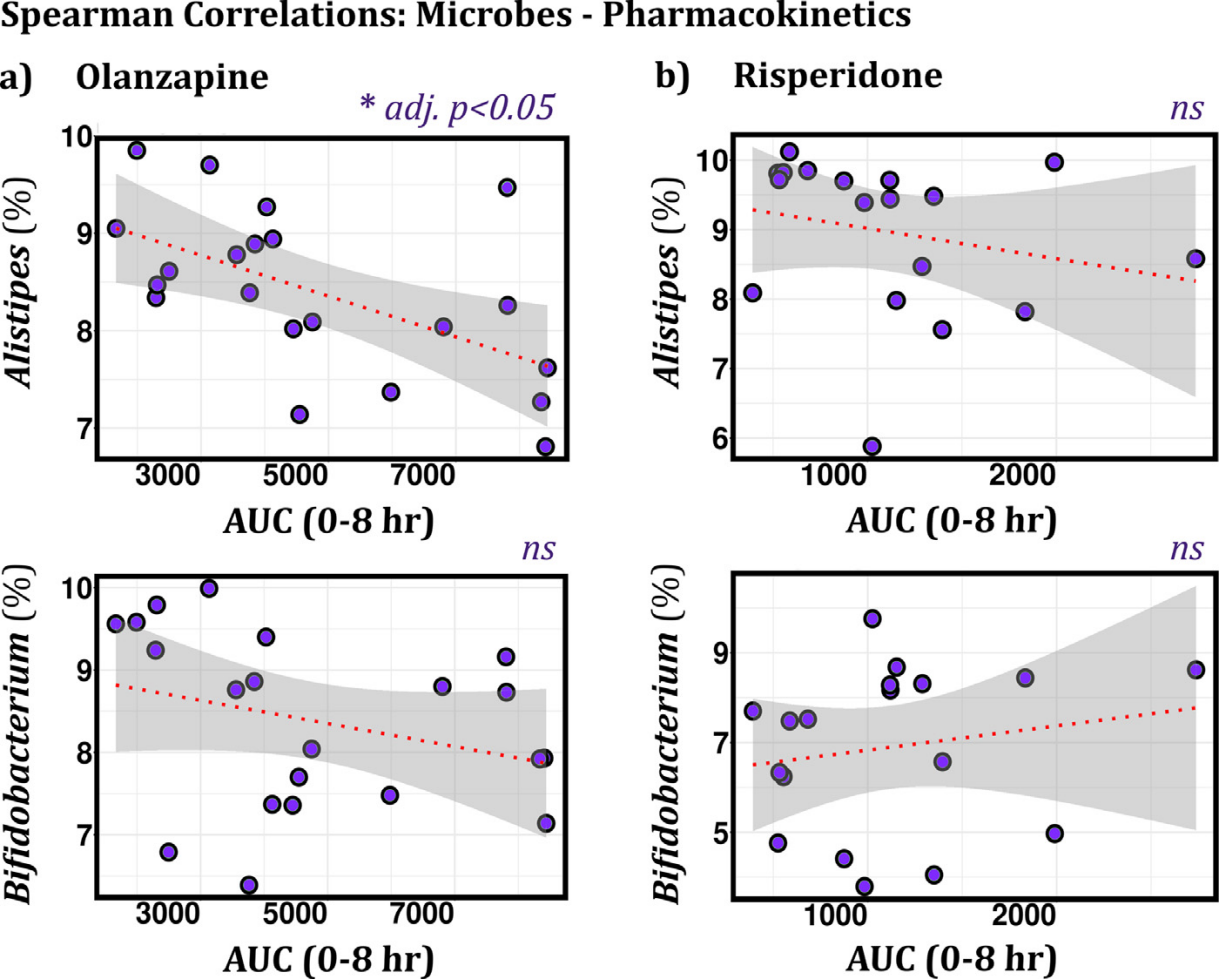
**Spearman correlations between AUC and the relative abundance of two bacteria in OLZ- and RISP-treated rats. (a)** In OLZ-treated rats, AUC significantly correlates with the relative abundance of *Alistipes* (*p* = 0.002245, adj *p* = 0.045, ρ = −0.64); *n* = 21. Bifidobacterium does not correlate with AUC. **(b)** In RISP-treated rats, no significant correlations are observed between AUC and bacterial abundance; *n* = 20. Data are normalized and CLR-transformed. *ns* = not significant.

After demonstrating that changes in the microbiome occurred in parallel with changes in the systemic absorption of OLZ (Fig. 4*a*), we compared whether the relative abundance of specific taxa was associated with pharmacokinetic parameters [including AUC_0-8h_, Cmax, Tmax, and CL] in both antipsychotic-receiving groups. In OLZ-treated rats, the relative abundance of *Alistipes* negatively and significantly correlated with AUC (Fig. 5). This correlation was not observed in RISP-treated rats. All other taxa, including *Bifidobacterium*, did not show significant correlations with pharmacokinetic-related parameters after adjusting for multiple testing (Fig. 5).

### 3.5 Neither intervention altered the levels of CYPs involved in the metabolism of antipsychotics

**Fig. 6:**
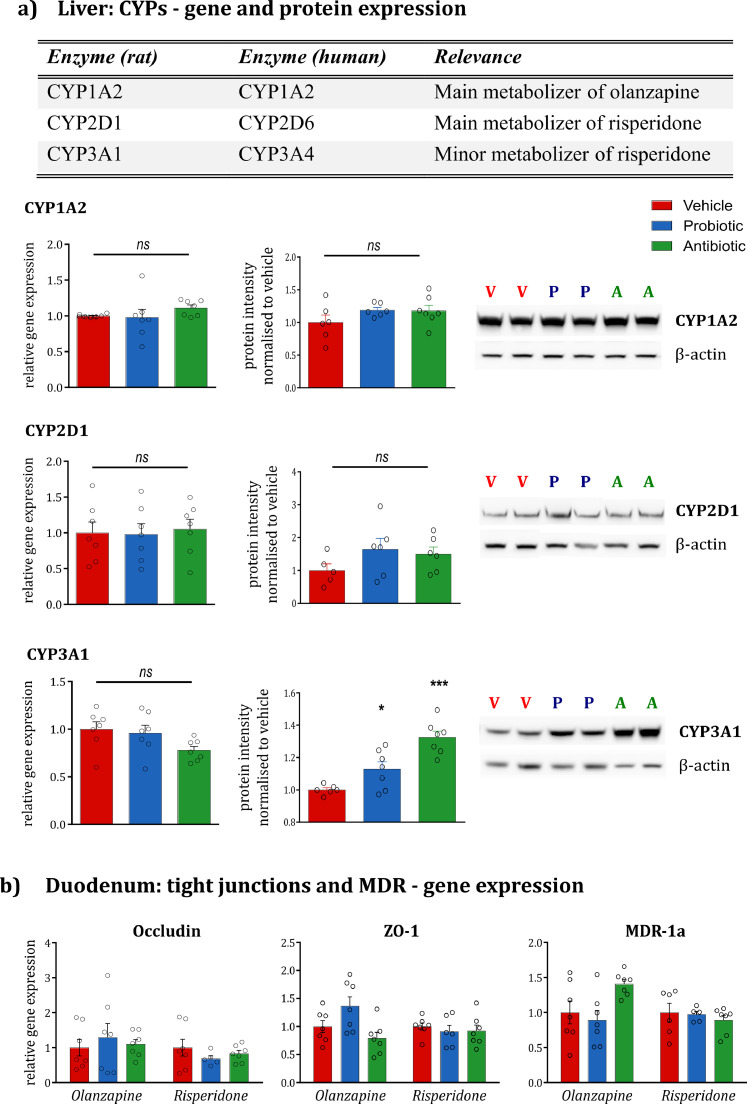
**The impact of microbiota-targeted interventions on hepatic and duodenal genes. (a)** Gene expression and protein levels of relevant CYPs in the liver. The literature-identified rat CYP enzymes equivalent to human CYPs implicated in the metabolism of OLZ and RISP are illustrated. Probiotic and antibiotic administration do not alter the gene expression and protein levels of *CYP1A2* and *CYP2D1* compared to the vehicle group. Both probiotics and antibiotics increase the protein levels of *CYP3A1* (One-way ANOVA F_(2;19)_=19.25, *p* < 0.001; post-hoc *p* = 0.049 for probiotic *vs* vehicle, *p* = 0.000 for antibiotic *vs* vehicle) without altering gene expression. The protein bands of two representative samples per group are shown. Data are expressed as mean + SEM *p < 0.05, ***p < 0.001, *ns*=not significant (*n* = 5–7/group). **(b)** Gene expression of relevant tight junctions and MDR in the duodenum**.** No significant differences are noted across groups. Data are expressed as mean + SEM (*n* = 5–7/group).

To test whether the probiotics or antibiotics had any direct effect on the hepatic expression of CYP enzymes relevant for the metabolism of antipsychotics, RT-qPCR was employed to examine the CYP gene expression at the transcript level. Interspecies comparison of CYPs isoforms present in rats and humans have been previously described (Fig. 6*a*) [32]. Neither the probiotic nor antibiotic treatment altered the hepatic mRNA expression of the rat-equivalent human isoenzymes implicated in the metabolism of OLZ and RISP (Fig. 6*a*). To further confirm the transcript findings at the protein level, Western blotting analysis of the same CYPs was performed. As shown in Fig. 6*a*, there was no difference in *CYP1A2* or *CYP2D1* protein in the livers of probiotic- and antibiotic-treated rats. Conversely, both microbiota-targeted interventions significantly upregulated *CYP3A1* protein, the magnitude of the effect more substantial in the case of the antibiotic-treated rats (*p* < 0.001 *vs* probiotic *p* < 0.05). As *CYP3A1* is, however, only a minor metaboliser of RISP, the significance of this finding is somewhat limited.

### 3.6 Neither microbiota-targeted intervention altered duodenal expression of tight junctions and *MDR-1A*

Previous studies have suggested that antibiotic administration can induce a dysregulation in intestinal barrier function [33,34], and the presence of the gut microbiota is important to maintain normal barrier function. For this reason, we assessed the gene expression of two key tight junctions, occludin and zonula occludens-1 (ZO-1) in the duodenum, the main site of absorption of xenobiotics. This was carried out to exclude the possibility that an antibiotic-induced barrier dysfunction in the duodenum might be responsible for the increased absorption of OLZ in the circulation. Neither probiotics nor antibiotics induced changes in the expression of tight junctions (Fig. 6*b*). The gene expression of multidrug resistance protein 1a (*MDR-1a*), a major efflux transporter in the intestinal lumen, was also assessed. The isoform 1a was selected in the duodenum because of its widespread distribution in this tissue compared to the isoform 1b [35]. Neither of the microbiota-targeted interventions, however, altered the expression of *MDR-1a* in the duodenum (Fig. 6*b*).

### 3.7 Antibiotic-induced microbiota depletion altered intestinal glucuronidation gene expression but not hepatic MDR-1A gene expression

**Fig. 7:**
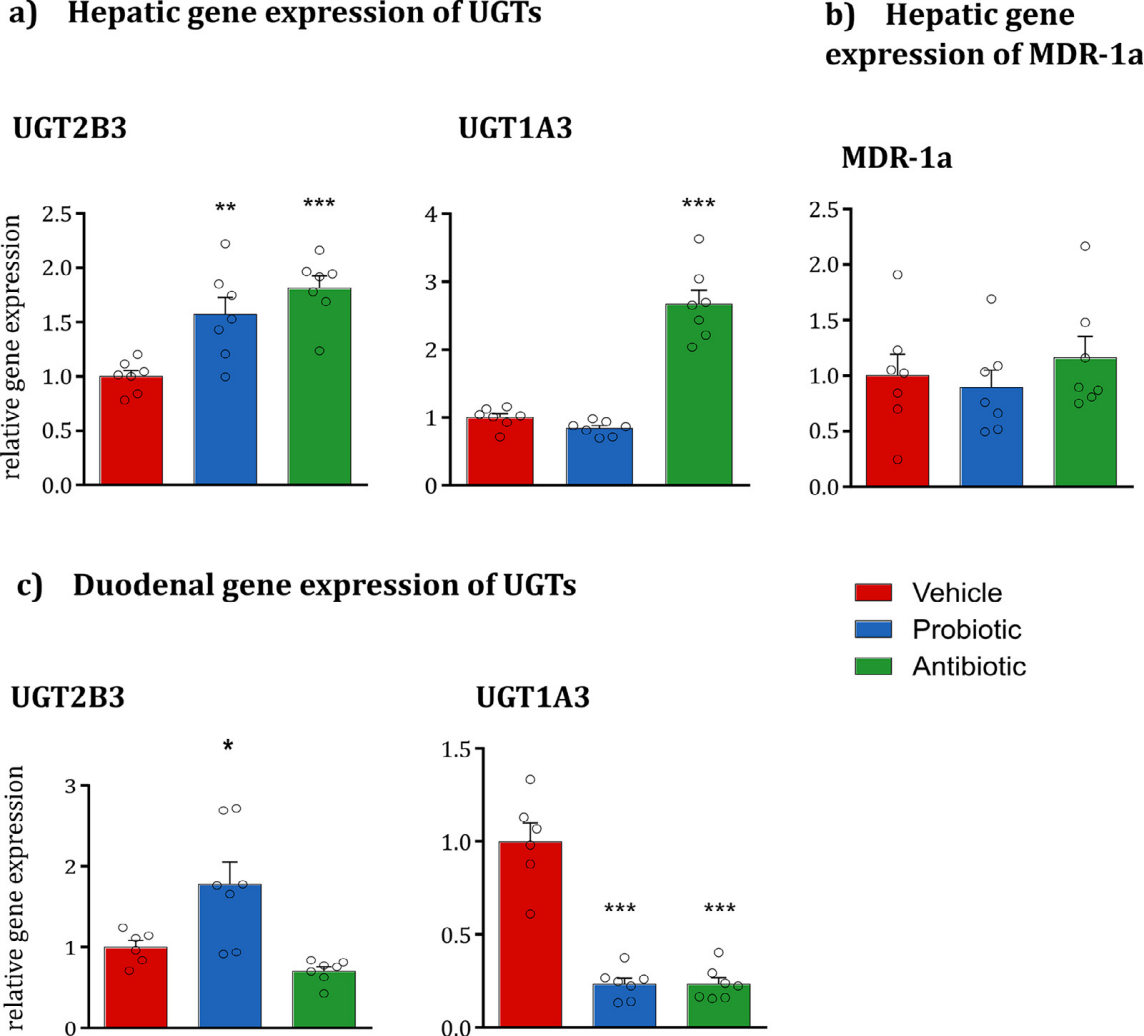
**Hepatic gene expression of UGTs and MDR-1a and duodenal gene expression of UGTs. (a)** Both probiotics and antibiotics increase the hepatic gene expression of *UGT2B3* (One-way ANOVA F_(2;20)_=13.14, *p* < 0.001; post-hoc *p* < 0.01 for probiotic *vs* vehicle and *p* < 0.001 for antibiotic *vs* vehicle). Antibiotics increase the hepatic gene expression of *UGT1A3* (One-way ANOVA F_(2;20)_=67.33, *p* < 0.001; post-hoc *p* < 0.001). *p < 0.05, **p < 0.05, ***p < 0.001. Data are expressed as mean + SEM (*n* = 6–7/group). **(b)** Both probiotics and antibiotics do not affect the hepatic gene expression of *MDR-1a*. Data are expressed as mean + SEM (*n* = 6–7/group). **(c)** Probiotic administration increases the duodenal gene expression of *UGT2B3* (One-way ANOVA F_(2;19)_=10.37, *p* < 0.01; post-hoc *p* < 0.05). Both probiotics and antibiotics decrease the duodenal gene expression of *UGT1A3* (One-way ANOVA F_(2;19)_=53.79, *p* < 0.001; post-hoc *p* < 0.001 for both groups *vs* vehicle). Data are expressed as mean + SEM (*n* = 6–7/group).

While OLZ undergoes an extensive first-pass effect, the major metabolic pathway involved in this first-pass effect is unclear. We aimed to assess if phase II metabolism, or possibly first-pass metabolism through UGT could be a contributory factor to the increased systemic absorption of OLZ in antibiotic-treated rats. There is limited evidence comparing species differences of UGT isoforms in rats and humans, especially in terms of substrate specificity. We, therefore, measured the transcript levels of *UGT2B3* and *UGT1A3* in the duodenum and liver, as surrogate rat isoforms predicted to be the most similar to the human *2B10* and *1A4,* respectively [36]. In the liver, antibiotics increased both UGT isoforms, while probiotics significantly increased the transcript levels of *UGT2B3* (Fig. 7*a*). None of the microbiota-targeted interventions, however, altered the expression of *MDR-1a* in the liver (Fig. 7*b*). In the duodenum, probiotic intake increased *UGT2B3* and decreased *UGT1A3*; while antibiotics reduced the transcript levels of *UGT1A3* (Fig. 7*c*). In addition, the protein levels of UGT1A were quantified both in the liver and in the duodenum (*Fig. S3*). Neither of the microbiota-targeted interventions significantly influenced the protein levels of the enzymatic family UGT1A at both body sites.

### 3.8 OLZ and RISP were not subject to fecalase-mediated metabolism

**Fig. 8:**
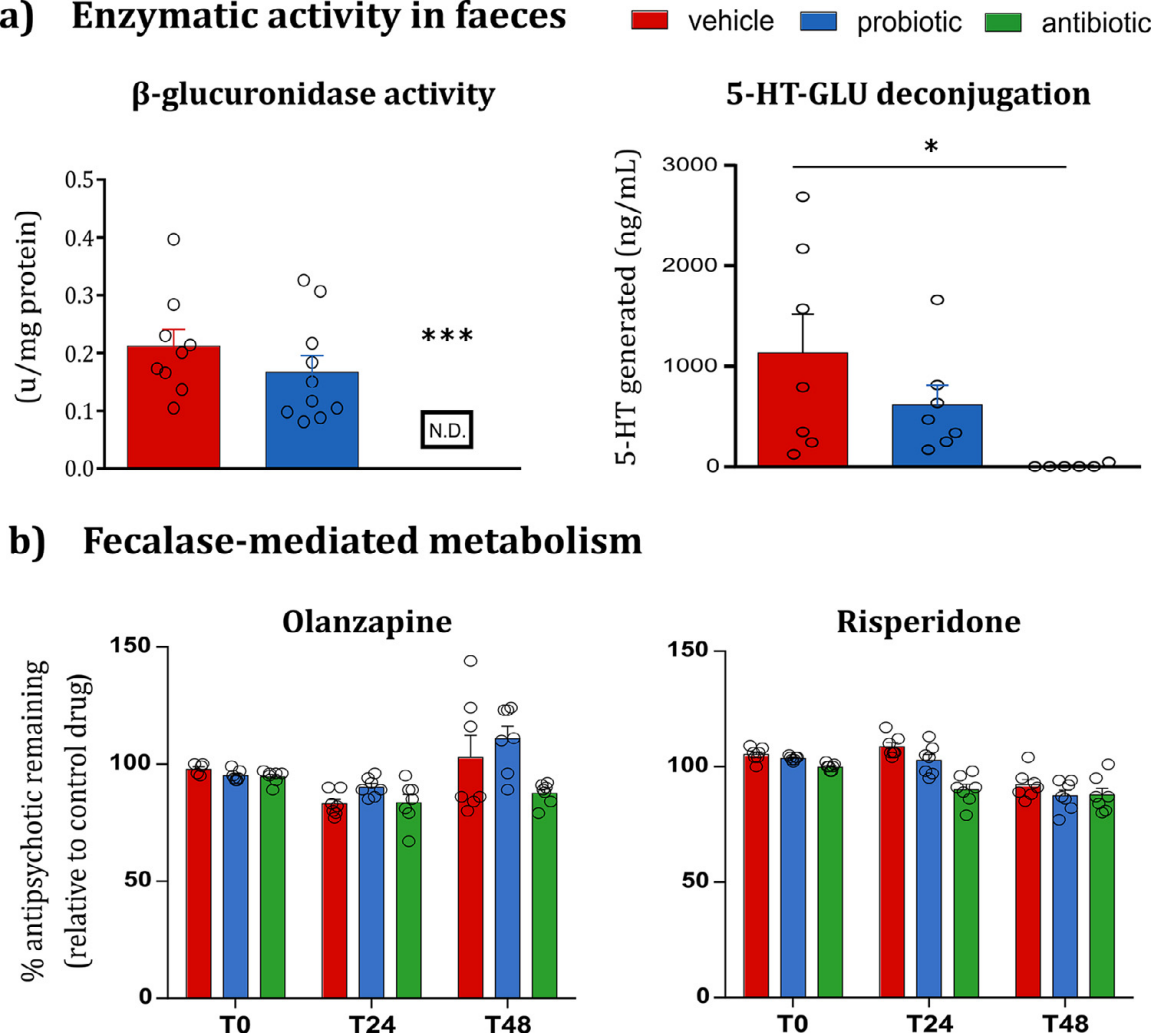
**Fecalase-mediated metabolism. (a)** Antibiotic-induced depletion of β-glucuronidase activity translates to altered deconjugation of 5-HT-GLU. Antibiotics downregulate β-glucuronidase enzymatic activity in fecalase (*vs* vehicle; *p* < 0.0001). The conversion of 5-HT-GLU to the parent compound, 5-HT, is significantly decreased in fecalase prepared from antibiotic-treated *vs* vehicle-treated rats (One-way ANOVA, *p* < 0.05). Probiotics do not alter the β-glucuronidase activity or the rate of deconjugation of 5-HT-GLU. Data are expressed as mean + SEM *p < 0.05 ***p < 0.001 (*n* =  9–10 for enzymatic readout; *n* = 7 for 5-HT-HPLC data). **(b)***Ex vivo* incubation of OLZ and RISP with fecalase. The amount of remaining OLZ and RISP is not altered after 48 h incubation with fecalase from antibiotic- and probiotic-treated rats compared to vehicle-treated rats. Data are expressed as mean + SEM, relative to the drug-only incubation controls (*n* = 6–7/group).

Fecalase, the enzyme fraction (i.e. cell free extract) of faeces [25], can be used as an *ex vivo* metabolism assay to study the metabolic activity of the gut microbiota, as well as xenobiotic metabolism by the intestinal bacteria. This strategy has previously elucidated the role of the gut microbiota in the metabolism of lovastatin, aspirin, and amlodipine. In this study, the activity of two microbial-derived enzymes was investigated as a surrogate readout of the metabolic activity of the gut microbiota. Antibiotic-induced microbiota depletion markedly decreased the expression of β-glucuronidase faecal enzymatic activity by the end of antibiotic treatment. This reduced β-glucuronidase activity was confirmed based on a significant reduction in the conversion of 5-HT-GLU to the parent compound, 5-HT, in antibiotic-treated rats relative to vehicle-treated rats. (Fig. 8*a*). Both microbiota-targeted interventions significantly reduced β-glucosidase activity relative to vehicle-only rats (*Fig. S2b*).

The probiotic treatment did not elicit a similar effect in β-glucuronidase. It is, therefore, tempting to speculate if different bacterial strains may express these enzymes. At this juncture, having shown the fecalase assay can successfully illustrate fecalase-mediated metabolism, the next step was to assess if OLZ or RISP were also subject to fecalase-mediated degradation. OLZ and RISP were incubated with fecalase from the vehicle-, probiotic- and antibiotic-treated rats for 48 h, and HPLC was used to estimate the remaining amount of the drug. Our data revealed that neither drug was degraded by fecalase (Fig. 8*b*).

## 4 Discussion

Pharmacomicrobiomics is an emerging field that explores the effects of microbiome variations on drug pharmacokinetics and pharmacodynamics [37,38]. While the vast metabolic capacity of the microbiome to metabolise a diverse array of drugs is clear, what remains unexplored is the extent to which changes in the microbiome can result in clinically significant changes in systemic drug levels.

In this study, two microbiota-targeted interventions were employed to assess whether the pharmacokinetic profile of two commonly prescribed antipsychotics, OLZ and RISP, might be influenced by changes in the microbiome. Our data reveal that the bioavailability of OLZ was increased 82% after a single oral dose following antibiotic-induced microbiome depletion, and provides evidence that the gut microbiome may play a role in modulating the oral bioavailability of OLZ. In contrast, the bioavailability of RISP was not influenced by microbiome perturbations. The study herein investigated several mechanisms through which the microbiota may influence the metabolism or absorption of OLZ. We hypothesised that OLZ may be directly metabolised by bacterial-derived enzymes or indirectly affected by microbiome-induced changes on host metabolism. Given the short half-life of the selected antibiotics and a 24 h wash out period before drug dosing, the risk of a carryover antibiotic effect on inhibiting host metabolism is considered minimal. The unchanged pharmacokinetics of RISP supports this rationale. Moreover, while certain antibiotics can induce host metabolic enzymes, we specifically chose those that have not been shown in literature to exert this effect. The most plausible explanation for the 1.8-fold increase in the systemic exposure to olanzapine in the microbiome-depleted group is a reduction in the first pass metabolism of this drug, linked to downregulation of duodenal UGT expression.

The gut microbiota has been previously linked to changes in the expression of phase I hepatic metabolism [39]. Neither the probiotic nor the antibiotic cocktail altered the hepatic expression of *CYP1A2* and *CYP2D1* implicated in the host metabolism of OLZ and RISP both at transcript and protein level (Fig. 6). The protein expression of *CYP3A1*, which was significantly increased in antibiotic-treated rats, has been previously increased by macrolide antibiotics in both rat and human hepatocytes [40]. *CYP3A1*, however, only plays a minor role in the metabolism of antipsychotics. Our results suggest therefore that the changes in pharmacokinetics following antibiotic treatment is not due to a direct inhibition of phase I hepatic pathways. VSL#3 administration did not impact the pharmacokinetics of either antipsychotic. Future studies employing different dosages and time-courses of the multi-strain probiotic cocktail, or examining each probiotic strain individually, are warranted.

First-pass intestinal- and liver-mediated metabolism can have an extensive impact on the bioavailability of UGT substrates in humans [41].  Glucuronidation via *UGT1A4*, and to a lesser extent *UGT2B10*, is the primary mode of OLZ metabolism; an effect that differentiates OLZ from RISP. Moreover, polymorphisms in these enzymes have been previously suggested as a potential source of inter-individual variability in OLZ metabolism and therapeutic response [42]. Interestingly, the activities of UGT1A3 and UGT1A4 can show up to 60-fold variation between individuals, increasing clinical risks such as significant variations of plasma concentrations and subsequent toxicity [43]. Hence, there remains an unmet need to identify the potential key drivers of this variability to guide and assist clinical dosage regulation in personalised medicine. The *in vivo* contribution of intestinal and hepatic glucuronidation in rats has been previously described in the literature, albeit a relatively unexplored area in pre-clinical research in comparison to the vast knowledge on human metabolism [44]. Nonetheless, the differential expression of UGT by antibiotic-induced microbiota depletion was investigated as a potential mechanistic explanation for the increased OLZ bioavailability. The significant downregulation of the *UGT1A4* equivalent isoform (*UGT1A3*) in the duodenum of antibiotic-treated rats hints at the potential for the microbiota to play a role in glucuronidation mediated by the intestine. Decreased metabolism of OLZ by the gastrointestinal enterocytes may hence be responsible for the increased levels of the drug reaching the systemic circulation. While pre-treatment with probiotics also significantly decreased the expression of *UGT1A3*, the accompanying probiotic-associated upregulation of the *UGT2B10* equivalent rat isoform (*UGT2B3*) may act as a compensatory metabolic route. In contrast to the duodenum, antibiotic-induced microbiota depletion significantly upregulated hepatic UGT expression (Fig. 7*b*). The higher expression of *UGT1A3* in the duodenum of rats in comparison to the liver may, however, limit the significance of this finding [36]. Neither microbiota manipulation induced a significant effect on the protein levels of the enzyme UGT1A in the duodenum and liver (Fig. S3). These results must be interpreted with caution, as there is some inconsistency in the literature regarding the relationship between protein levels and functional enzymatic activity [45]. In cases where protein levels do not match the transcript levels, several factors could be responsible for this discrepancy, including protein stability, post-transcriptional regulations and degradation rate of the mRNA [46]. In addition, previous studies in humans have reported not only a divergence between UGT1A mRNA and protein levels [47], [48], [49], but also a high degree of variability in UGT mRNA and protein expression within different tissues and different individuals [47,50]. In addition, considering the wide range of isoforms detected from the antibody UGT1A, a microbiota-mediated effect on the isoform 1A3 cannot be completely ruled out. Due to the inter-species differences in glucuronidation, and the limited evidence of specific rat UGT enzymes implicated in the metabolism of OLZ, care must be exercised in the interpretation and extrapolation of the results to humans [51].

Evidence suggests that antibiotics can induce a barrier dysfunction in the GI tract [33,34]. With increased levels of OLZ in the blood of antibiotic-treated rats, a disruption in intestinal permeability might have been the cause of an increased absorption at the duodenum level. However, according to the gene expression of two tight junction proteins, occludin and ZO-1, antibiotics did not alter duodenal permeability.

To elucidate whether bacterial-derived enzymes could directly metabolise OLZ, we next utilised fecalase as an *ex vivo* screening platform for gut microbiota-mediated drug metabolism. Antibiotic treatment significantly depleted the enzymatic activity of two microbial enzymes, with the activity of β-glucuronidase falling to levels below the limit of detection of the assay (Fig. 8*a*). The suppressed metabolic activity significantly reduced the deconjugation of 5-HT-GLU to 5-HT after brief exposure to fecalase (i.e., 1 h incubation). This reduced metabolic activity, however, did not translate to altered metabolism of OLZ even after incubation for a total of 48 h (Fig. 8*b*). Suppression of the metabolic activity of the gut microbiota has been previously linked to increased bioavailability of both aspirin and amlodipine in rats pre-treated with a single dose of ampicillin [52,53]. In both studies, fecalase from antibiotic-treated rats reduced the formation of a drug metabolite. Thus, the fecalase-mediated metabolism of OLZ could be further investigated as a potential mechanism by comparing the formation rate of OLZ metabolites in fecalase from antibiotic-treated rats versus vehicle-only rats. Further studies could continue to investigate the direct microbial metabolism of OLZ by testing the hypothesis that fecalase, specifically the microbial-derived enzyme β-glucuronidase, could convert a glucuronide-conjugated metabolite of OLZ (OLZ 10-N glucuronide or OLZ 4-N glucuronide) into the parent compound through a similar *ex vivo* incubation assay. Furthermore, OLZ may be subject to bacterial-mediated metabolism higher up the rat intestinal region, albeit an area associated with lower microbial colonisation. A crude enzymatic fraction isolated from the upper small intestine has been previously investigated for the probiotic-mediated metabolism of acetaminophen [54].

Across 156 genera detected by 16S sequencing, *Alistipes* only correlated with the bioavailability (i.e., AUC) of OLZ- but not RISP-treated rats, suggesting that this specific bacterium may play a role in the pharmacokinetic alterations of OLZ. Intriguingly, the genus *Alistipes* has been previously linked to patient response to chemotherapy. Specifically, the abundance of *Alistipes* correlated positively with the immunotherapy-induced production of TNF in conventional mice [55]. Administration of an antibiotic cocktail (vancomycin, imipenem, and neomycin in drinking water) significantly disrupted the microbiome, impaired immunotherapy efficacy, and, therefore, TNF production [55]. Moreover, *Alistipes indistinctus* (DSM 22520) was recently identified as one bacterial strain linked to the chemical modification of approximately 40 drugs; half of these substrate drugs were greater than 80% metabolised [4]. Interestingly, the authors showed greater than 50% of RISP was metabolised after 12 h incubation with this bacterial strain. Our data, however, did not find an association between RISP and *Alistipes.* Although the evidence on the role played by specific taxa on drug response is still limited, the research by Iida, and Zimmermann, and colleagues’ sheds light upon the potential role of *Alistipes* in drug efficacy.

The results of this initial proof-of-concept study proposes that antibiotic-depletion of olanzapine glucuronidation on first pass through the small intestine as the plausible mechanism underpinning the 1.8-fold increase in olanzapine bioavailability. Further unravelling the mechanistic basis behind the overall impact of microbiota depletion on drug pharmacology is, however, warranted and may provide valuable insight in this field. A follow-on study where these antipsychotics are administered intravenously could offer a strategy to bypass intestinal first-pass metabolism and clarify the magnitude of the antibiotic-induced microbiota depletion on drug absorption. This study is not, however, without limitations. The expression and protein levels of host metabolic genes were determined using whole liver or duodenal samples, whereas specific microsomal expression would provide further insights. The activity of β-glucuronidase and β-glucosidase enzymes was studied primarily as a surrogate readout of the metabolic activity of the gut microbiota; we cannot discount that other bacterial enzymes may play a direct role in OLZ metabolism. The identification of specific enzymes implicated in the metabolism of OLZ and RISP, and the impact of these microbiota-targeted interventions on such enzymes, is warranted.

While this study suggests that chronic antibiotic use may lead to elevated olanzapine levels, a complex relationship between drug pharmacokinetics and pharmacodynamics exists. As mentioned, our previous research illustrated antibiotic-induced depletion of the gut microbiota can dissipate the metabolic side-effects associated with OLZ, an effect that could be presumed contrary to these pharmacokinetic results. While the 1.8-fold increase in the systemic availability of OLZ may increase the risk of dose-limiting side-effects, including somnolence and dizziness and metabolic dysfunction [56,57], the overall clinical relevance may be dependent on specific patient-related factors including age, hepatic function and concomitantly taken medication. Nonetheless, elevated OLZ levels have been linked to an increased risk of developing diabetes, peripheral oedema and postural hypotension and warrant consideration in clinical practice [58]. Clinically, antibiotic-related interactions with OLZ have, thus far, been linked to the direct inhibition of CYP1A2; the co-prescription of ciprofloxacin may necessitate a reduction in OLZ dosage to minimize adverse effects. The results herein provide further scope to consider the impact of antibiotic-induced effects on OLZ therapy.

## Contributors

SC, JW, TGD, NPH, GC, JFC, and BTG designed the experiment. SC and JW conducted the experiments and prepared the figures. AVG and AVZ conducted western blotting for the protein UGT1A. SC, JW, AVG, NPH, GC, JFC, and BTG performed literature search and participated in data interpretation. SC, JW, GC, CRS, and FF were involved in data collection. SC, JW, BTG, CRS, FF, and CS performed data analysis. SC and JW wrote the initial draft of the manuscript. All authors critically revised the manuscript and approved the final version.

## Data sharing

Study protocol and all data collected for the study, including raw data and data analysis will be made available to others upon request. All data will be available upon publication of the manuscript, by contacting the corresponding author. Data will be made available after approval of a proposal and with a signed data access agreement.

## Declaration of Competing Interest

BTG has spoken at events sponsored by AbbVie. CS receives funding from 4D Pharma, Dupont and Nutricia. GC has spoken at events sponsored by Janssen Ireland and Probi, and receives funding from Pharmavite. JFC has spoken at events sponsored by Janssen Ireland, Neuropharmex, Mead Johnson, Friesland Campina, has been a consultant for Nestle & Alkermes and receives funding from Mead Johnson, Cremo, 4D Pharma, Dupont, GSK, Suntory Wellness, Pharmavite, and Nutricia. NPH has spoken at events sponsored by Johnson & Johnson and Coloplast. TGD has spoken at events sponsored by Servier, Lundbeck, Janssen, AstraZeneca and receives funding from Mead Johnson, Cremo, 4D Pharma, Dupont, GSK, Suntory Wellness, Pharmavite, and Nutricia.

## Appendix Supplementary materials

Image, application 1

Image, application 2

Image, application 3
