# Supplementary material for: The gut microbiome influences the bioavailability of olanzapine in rats
Source: eBioMedicine. 2021 Apr 2;66:103307. doi: 10.1016/j.ebiom.2021.103307 (PMC8047500; doi:10.1016/j.ebiom.2021.103307)
Supplement: Supplementary file 2 [file mmc2.pdf]

SUPPLEMENTAL FIGURES

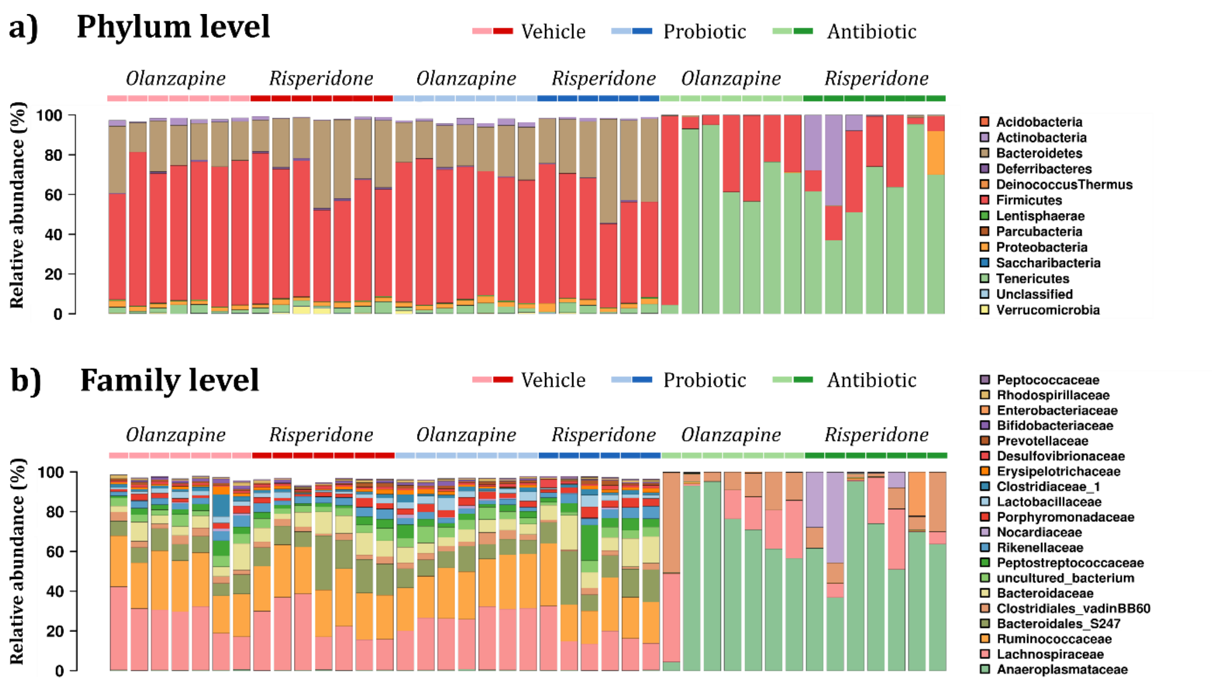

Figure S1. **Bar charts representing the taxa abundance at the phylum (a) and family (b) levels**. The 20 most abundant taxa are shown.

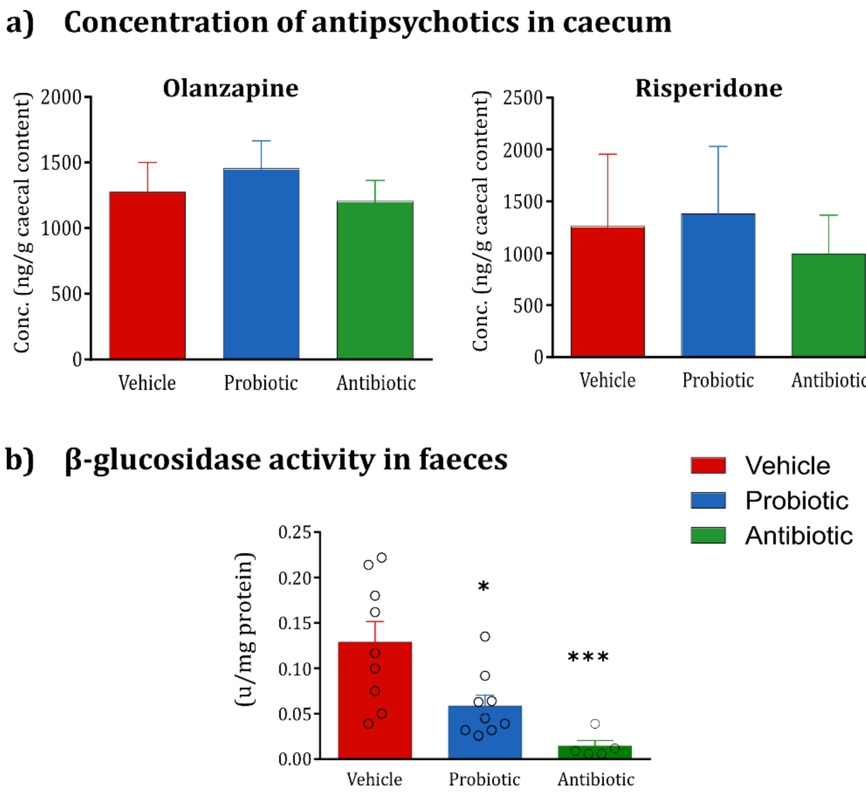

Figure S2. Caecal concentration of antipsychotics and faecal enzymatic activity. (a) Antipsychotic concentration in the caecum after oral administration in rats pre-treated with vehicle, probiotic or antibiotic. Neither antibiotic or probiotic treatment significantly altered the concentration of olanzapine (n=7) or risperidone (n=5-7) in the caecum. Data are expressed as mean + SEM. **(b)** β-glucosidase activity in rat faecal samples from vehicle, probiotic and antibiotic-treated rats. Data are expressed as mean + SEM. *p<0.05 ***p<0.001 (n=8).

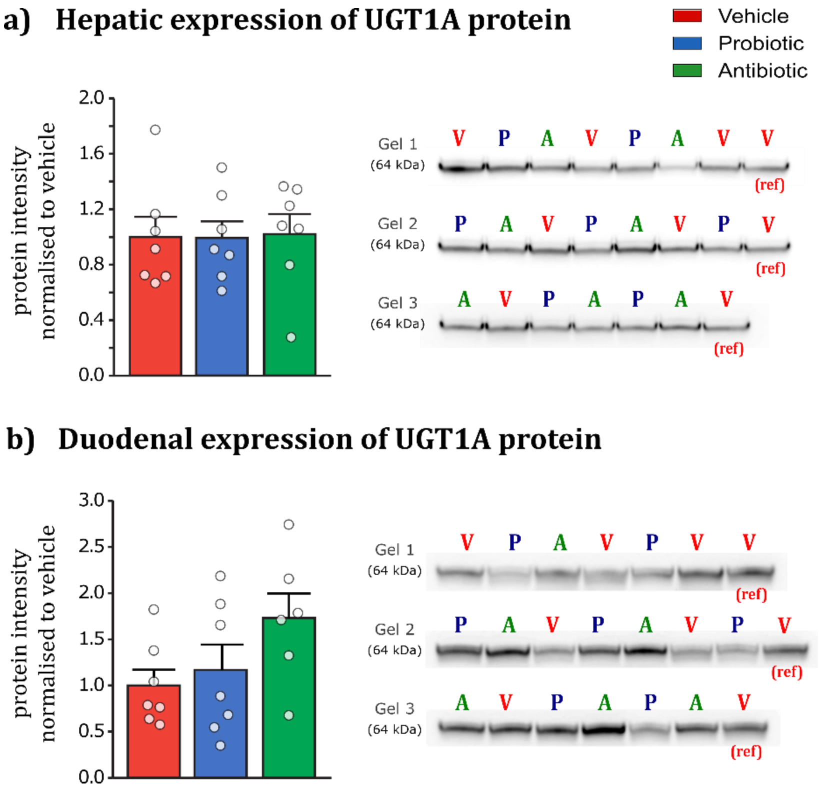

Figure S3. Hepatic (a) and duodenal (b) UGT1A protein expression in rats pre-treated with vehicle, probiotic or antibiotic. Probiotic and antibiotic administration do not alter the protein expression of UGT1A family. Data are expressed as mean + SEM, n=6-7/group.

SUPPLEMENTAL TABLES

Table S1. Statistics values for relative abundance of bacterial PHYLA in each treatment group (probiotic or antibiotic) as compared to the vehicle. *$p<0.05$, Mann-Whitney U test, Benjamini-Hochberg critical value [(i/m)Q] with i=rank; m=total number of tests; Q (false-discovery rate)=0.2. N=13-14/group. Red indicates an increase, blue a decrease compared to the vehicle group. ns=not significant

**Phylum**	**Probiotic**		**Antibiotic**	
	p value	***(i/m)·Q***	***p value***	***(i/m)·Q***
Firmicutes	ns	ns	0.00	0.0181 *
Verrucomicrobia	ns	ns	0.00	0.0363 *
Bacteroidetes	ns	ns	0.00	0.0545 *
Saccharibacteria	ns	ns	0.00	0.0727 *
Tenericutes	ns	ns	0.00	0.0909 *
Deferribacteres	ns	ns	0.00	0.1090 *
Proteobacteria	ns	ns	0.00	0.1454 *
Lentisphaerae	ns	ns	0.00	0.1636 *
Unclassified	ns	ns	0.00	0.1818 *
Actinobacteria	ns	ns	0.02	0.2

Table S2. Statistics values for relative abundance of bacterial FAMILIES in each treatment group (probiotic or antibiotic) as compared to the vehicle. *$p<0.05$, Mann-Whitney U test, Benjamini-Hochberg critical value [(i/m)Q] with i=rank; m=total number of tests; Q (false-discovery rate)=0.2. N=13-14/group. Red indicates an increase, blue a decrease compared to the vehicle group. ns=not significant.

**Family**	**Probiotic**		**Antibiotic**	
	p value	***(i/m)·Q***	***p value***	***(i/m)·Q***
Desulfovibrionaceae	ns	ns	0.00	0.004878 *
Rhodospirillaceae	ns	ns	0.00	0.009756 *
Ruminococcaceae	ns	ns	0.00	0.014634 *
Peptostreptococcaceae	ns	ns	0.00	0.019512 *
Lactobacillaceae	ns	ns	0.00	0.02439 *
Caldicoprobacteraceae	ns	ns	0.00	0.029268 *
Christensenellaceae	0.04	0.019512 *	0.00	0.034146 *
Flavobacteriaceae	ns	ns	0.00	0.039024 *
Clostridiaceae_1	ns	ns	0.00	0.043902 *
Bacteroidales_S24_7_group	ns	ns	0.00	0.04878 *
uncultured_rumen_bacterium	ns	ns	0.00	0.053659 *
Peptococcaceae	ns	ns	0.00	0.058537 *
Streptococcaceae	0.01	0.009756 *	0.00	0.063415 *
Bifidobacteriaceae	ns	ns	0.00	0.068293 *
Victivallaceae	ns	ns	0.00	0.073171*
Prevotellaceae	ns	ns	0.00	0.078049 *
Anaeroplasmataceae	ns	ns	0.00	0.082927 *
Bacteroidaceae	ns	ns	0.00	0.087805 *
Micrococcaceae	ns	ns	0.00	0.092683 *
Porphyromonadaceae	0.01	0.004878	0.00	0.097561 *
Eubacteriaceae	ns	ns	0.00	0.102439 *
Coriobacteriaceae	ns	ns	0.00	0.107317 *
Family_XIII	ns	ns	0.00	0.112195 *
Unknown_Family	ns	ns	0.00	0.117073 *
Deferribacteraceae	ns	ns	0.00	0.121951 *
Erysipelotrichaceae	ns	ns	0.00	0.126829 *
uncultured_organism	ns	ns	0.00	0.131707 *
Alcaligenaceae	ns	ns	0.00	0.136585 *
Verrucomicrobiaceae	ns	ns	0.00	0.141463 *
Rikenellaceae	ns	ns	0.00	0.146341 *
uncultured_bacterium	ns	ns	0.00	0.15122 *
Unclassified	ns	ns	0.00	0.156098 *
Clostridiales_vadinBB60_group	ns	ns	0.00	0.160976 *
Pasteurellaceae	ns	ns	0.00	0.165854 *
Thermoanaerobacteraceae	ns	ns	0.01	0.170732 *
Lachnospiraceae	ns	ns	0.01	0.17561 *
Staphylococcaceae	0.03	0.014634 *	ns	ns

Table S3. Statistics for relative abundance of bacterial <u>GENERA</u> in each treatment group as compared to the vehicle. *$p<0.05$, Mann-Whitney U test, Benjamini-Hochberg critical value [(i/m)Q] with i =rank; m=total number of tests; Q (false-discovery rate)=0.2. N=13-14/group. Values in red bold are significantly different from vehicle. Pink highlighted text indicates genera that are increased relative to vehicle whilst blue highlighted text indicates genera that are decreased relative to vehicle.

Probiotic		
**genus**	***p***	**(i/m)·Q**
Streptococcus	0.012	**0.0017094**
Odoribacter	0.033	**0.0034188**
Anaerofilum	0.033	**0.0051282**
Incertae_Sedis	0.043	**0.0068376**
Marvinbryantia	0.043	**0.008547**
Aerococcus	0.043	**0.0102564**
Erysipelatoclostridium	0.043	**0.0119658**
Eisenbergiella	0.048	**0.0136752**
Christensenellaceae_R_7_group	0.054	0.0153846
Staphylococcus	0.061	0.017094
Lachnospiraceae_UCG_008	0.061	0.0188034
Acetitomaculum	0.061	0.0205128
Ruminococcaceae_UCG_005	0.085	0.0222222
Eubacterium_coprostanoligenes_	0.094	0.0239316
Ruminiclostridium	0.094	0.025641
Peptoclostridium	0.094	0.0273504
Gordonibacter	0.094	0.0290598
Coriobacteriaceae_UCG_002	0.105	0.0307692
Ruminiclostridium_6	0.128	0.0324786
Alloprevotella	0.141	0.034188
Tyzzerella	0.141	0.0358974
Enterorhabdus	0.155	0.0376068
Alistipes	0.155	0.0393162
Lachnospiraceae_FCS020_group	0.169	0.0410256
Coprococcus_3	0.169	0.042735
Jeotgalicoccus	0.169	0.0444444
Asaccharobacter	0.169	0.0461538
Roseburia	0.185	0.0478632
Ruminococcus_1	0.22	0.0495726
Intestinibacter	0.22	0.0512821
Anaerovorax	0.239	0.0529915
Lachnoclostridium	0.239	0.0547009
unidentified	0.259	0.0564103
Ruminococcaceae_UCG_003	0.28	0.0581197
Lachnospiraceae_UCG_010	0.302	0.0598291
Blautia	0.302	0.0615385
Butyricimonas	0.302	0.0632479
Lachnospiraceae_NK4A136_grou	0.325	0.0649573
Ruminococcaceae_NK4A214_gro	0.325	0.0666667
Mogibacterium	0.325	0.0683761
Butyrivibrio	0.325	0.0700855
Enterococcus	0.325	0.0717949
Ruminococcaceae_UCG_014	0.325	0.0735043
Ruminiclostridium_9	0.35	0.0752137
Mucispirillum	0.35	0.0769231
Caldicoprobacter	0.375	0.0786325
Unclassified_uncultured_bacteriu	0.402	0.0803419
Coprococcus_1	0.402	0.0820513

Probiotic contd.		
**genus**	***p***	**(i/m)·Q**
Lactobacillus	0.43	0.0837607
Pasteurella	0.43	0.0854701
Bilophila	0.43	0.0871795
Bifidobacterium	0.458	0.0888889
Unclassified_uncultured_organism	0.458	0.0905983
Allobaculum	0.458	0.0923077
Papillibacter	0.458	0.0940171
Gelria	0.488	0.0957265
Eubacterium_ruminantium_group	0.488	0.0974359
Ruminiclostridium_1	0.488	0.0991453
Intestinimonas	0.488	0.1008547
Parasutterella	0.519	0.1025641
Family_eighth_UCG_001	0.519	0.1042735
Candidatus_Arthromitus	0.519	0.1059829
Eubacterium_nodatum_group	0.55	0.1076923
Lachnospiraceae_UCG_001	0.55	0.1094017
Acetanaerobacterium	0.55	0.1111111
Acetatifactor	0.55	0.1128205
Lactonifactor	0.55	0.1145299
Lachnospiraceae_UCG_005	0.583	0.1162393
Ruminococcus_2	0.583	0.1179487
uncultured_rumen_bacterium	0.583	0.1196581
Lachnospiraceae_NC2004_group	0.616	0.1213675
Unclassified_unidentified	0.616	0.1230769
Paraprevotella	0.616	0.1247863
Bacteroides	0.616	0.1264957
Turicibacter	0.616	0.1282051
Enterobacter	0.65	0.1299145
Prevotellaceae_UCG_001	0.65	0.1316239
Unclassified	0.65	0.1333333
Akkermansia	0.685	0.1350427
Rothia	0.72	0.1367521
Desulfovibrio	0.72	0.1384615
Thalassospira	0.72	0.1401709
Ruminococcaceae_UCG_010	0.756	0.1418803
Candidatus_Saccharimonas	0.756	0.1435897
Clostridium_sensu_stricto_1	0.793	0.1452991
Unclassified_uncultured_Mollicut	0.793	0.1470085
Anaerotruncus	0.83	0.1487179
Rikenella	0.83	0.1504274
Hydrogenoanaerobacterium	0.83	0.1521368
Natranaerovirga	0.83	0.1538462
Oscillibacter	0.83	0.1555556
Eubacterium_oxidoreducens_grou	0.867	0.157265
Ruminococcaceae_UCG_009	0.867	0.1589744
Lachnospiraceae_NK4B4_group	0.867	0.1606838
Barnesiella	0.867	0.1623932
Flavonifractor	0.867	0.1641026
Family_eighth_AD3011_group	0.867	0.165812
Unclassified_uncultured_rumen_b	0.867	0.1675214
Eubacterium_ventriosum_group	0.905	0.1692308
Candidatus_Soleaferrea	0.905	0.1709402
Asteroleplasma	0.905	0.1726496
Victivallis	0.943	0.174359
Eubacterium_brachy_group	0.943	0.1760684
Ruminiclostridium_5	0.943	0.1777778
Parabacteroides	0.943	0.1794872

Probiotic contd.		
**genus**	***p***	**(i/m)·Q**
Ruminococcaceae_UCG_013	0.943	0.1811966
Anaerofustis	0.981	0.182906
Lachnospiraceae_UCG_004	0.981	0.1846154
Lachnospiraceae_AC2044_group	0.981	0.1863248
uncultured	0.981	0.1880342
Sporobacter	1	0.1897436
Peptococcus	1	0.191453
Rikenellaceae_RC9_gut_group	1	0.1931624
Anaeroplasma	1	0.1948718
Tyzzerella_3	1	0.1965812
Senegalimassilia	1	0.1982906
Shuttleworthia	1	0.2

Antibiotic		
genus	*p*	(i/m)·Q
Lachnospiraceae_NC2004_group	0	**0.0017094**
Coriobacteriaceae_UCG_002	0	**0.0034188**
Eubacterium_ventriosum_group	0	**0.0051282**
Lachnospiraceae_UCG_005	0	**0.0068376**
Ruminiclostridium_9	0	**0.008547**
Eubacterium_coprostanoligenes_	0	**0.0102564**
Incertae_Sedis	0	**0.0119658**
Roseburia	0	**0.0136752**
Ruminococcus_1	0	**0.0153846**
Lactobacillus	0	**0.017094**
Victivallis	0	**0.0188034**
Bifidobacterium	0	**0.0205128**
Eubacterium_brachy_group	0	**0.0222222**
Marvinbryantia	0	**0.0239316**
Eubacterium_oxidoreducens_grou	0	**0.025641**
Ruminococcaceae_UCG_009	0	**0.0273504**
Enterorhabdus	0	**0.0290598**
Clostridium_sensu_stricto_1	0	**0.0307692**
Unclassified_uncultured_organism	0	**0.0324786**
Lachnospiraceae_UCG_010	0	**0.034188**
Ruminococcus_2	0	**0.0358974**
Eubacterium_ruminantium_group	0	**0.0376068**
Anaerovorax	0	**0.0393162**
Ruminococcaceae_UCG_003	0	**0.0410256**
Anaerofustis	0	**0.042735**
Lachnospiraceae_NK4A136_grou	0	**0.0444444**
Ruminococcaceae_UCG_010	0	**0.0461538**
Parasutterella	0	**0.0478632**
Ruminococcaceae_NK4A214_gro	0	**0.0495726**
Streptococcus	0	**0.0512821**
Mogibacterium	0	**0.0529915**
Akkermansia	0	**0.0547009**
Butyrivibrio	0	**0.0564103**
Rothia	0	**0.0581197**
Ruminiclostridium_5	0	**0.0598291**
Lachnospiraceae_NK4B4_group	0	**0.0615385**
Eubacterium_nodatum_group	0	**0.0632479**
Barnesiella	0	**0.0649573**
Anaerotruncus	0	**0.0666667**
Christensenellaceae_R_7_group	0	**0.0683761**
Peptococcus	0	**0.0700855**
Parabacteroides	0	**0.0717949**
Rikenella	0	**0.0735043**
Bilophila	0	**0.0752137**
unidentified	0	**0.0769231**
Lachnospiraceae_UCG_004	0	**0.0786325**
Lachnospiraceae_AC2044_group	0	**0.0803419**
Hydrogenoanaerobacterium	0	**0.0820513**
Paraprevotella	0	**0.0837607**
Lachnospiraceae_FCS020_group	0	**0.0854701**
uncultured_rumen_bacterium	0	**0.0871795**
Lachnospiraceae_UCG_008	0	**0.0888889**
Alloprevotella	0	**0.0905983**
Desulfovibrio	0	**0.0923077**
Natranaerovirga	0	**0.0940171**
Ruminococcaceae_UCG_005	0	**0.0957265**
Blautia	0	**0.0974359**

Antibiotic contd.		
genus	*p*	(i/m)·Q
Lachnospiraceae_UCG_001	0	**0.0991453**
Eisenbergiella	0	**0.1008547**
Family_XIII_UCG_001	0	**0.1025641**
Rikenellaceae_RC9_gut_group	0	**0.1042735**
Ruminiclostridium_1	0	**0.1059829**
Ruminiclostridium	0	**0.1076923**
Acetitomaculum	0	**0.1094017**
Coprococcus_3	0	**0.1111111**
Ruminiclostridium_6	0	**0.1128205**
Unclassified_uncultured_bacteriu	0	**0.1145299**
Candidatus_Soleaferrea	0	**0.1162393**
Acetatifactor	0	**0.1179487**
Intestinimonas	0	**0.1196581**
Anaeroplasma	0	0.1213675
Bacteroides	0	**0.1230769**
Allobaculum	0	**0.1247863**
Odoribacter	0	**0.1264957**
Flavonifractor	0	**0.1282051**
Lachnoclostridium	0	**0.1299145**
Peptoclostridium	0	**0.1316239**
Erysipelatoclostridium	0	**0.1333333**
Tyzzerella	0	**0.1350427**
Anaerofilum	0	**0.1367521**
Ruminococcaceae_UCG_013	0	**0.1384615**
Family_XIII_AD3011_group	0	**0.1401709**
Unclassified_uncultured_rumen_b	0	**0.1418803**
Butyricimonas	0	**0.1435897**
Caldicoprobacter	0	**0.1452991**
Tyzzerella_3	0	**0.1470085**
Asteroleplasma	0	**0.1487179**
Asaccharobacter	0	**0.1504274**
Alistipes	0	**0.1521368**
Ruminococcaceae_UCG_014	0	**0.1538462**
uncultured	0	**0.1555556**
Senegalimassilia	0	**0.157265**
Shuttleworthia	0	**0.1589744**
Papillibacter	0	**0.1606838**
Turicibacter	0	**0.1623932**
Gordonibacter	0	**0.1641026**
Candidatus_Saccharimonas	0	**0.165812**
Prevotellaceae_UCG_001	0	**0.1675214**
Oscillibacter	0	**0.1692308**
Thalassospira	0	**0.1709402**
Coprococcus_1	0	**0.1726496**
Mucispirillum	0	**0.174359**
Unclassified	0	**0.1760684**
Pasteurella	0.001	**0.1777778**
Sporobacter	0.001	**0.1794872**
Acetanaerobacterium	0.003	**0.1811966**
Candidatus_Arthromitus	0.003	**0.182906**
Lactonifactor	0.003	**0.1846154**
Gelria	0.008	**0.1863248**
Unclassified_uncultured_Mollicut	0.056	0.1880342
Enterobacter	0.062	0.1897436
Enterococcus	0.125	0.191453
Staphylococcus	0.178	0.1931624
Unclassified_unidentified	0.21	0.1948718

Antibiotic contd.		
**genus**	***p***	**(i/m)·Q**
Intestinibacter	0.541	0.1965812
Jeotgalicoccus	0.769	0.1982906
Aerococcus	1	0.2

Table S4. List of SybrGreen probes used in the study. Provider company: Eurofins Genomics.

Gene	Common gene name	Sequence (5' → 3') left	Sequence (5' → 3') right
Actin β	Actin beta	CCCGCGAGTACAACCTTCT	CGTCATCCATGGCGAACT
Occludin	Occludin	GCTATGAAACCGACTACACGACA	ACTCTCCAGCAACCAGCATCT
ZO-1	Zonula occludens-1	AGGCTATTTCCAGCGTTTTGA	AATCCTGGTGGTGGTACTTGC
MDR-1a	Multidrug resistance protein 1a	GAAAGGAATTTACTTCAAACTTGTCA	CACAAGCTTCATTTCCTAATTCAA
CYP1A2	Cytochrome P450 Family 1 Subfamily A Member 2	AATGACATCTTTGGAGCTGGAT	GGGCTCTGTCACAAGTAGCA
CYP3A1	Cytochrome P450 Family 3 Subfamily A Member 1	CATGTCTGAGGATGAAGAATGG	TGTCTCATGAGGGGGAACAT
CYP2D1	Cytochrome P450 Family 2 Subfamily D Member 1	GAGTGTTGGCCAGTGGTCTT	CAGCAGCTCCATGTCTGC

SUPPLEMENTAL METHODS

Caecal microbiota composition

Caecum was harvested, snap frozen and stored at -80ºC prior to the analysis.

- *Caecal content DNA extraction*

DNA extraction was performed using the QIAmp Fast DNA Stool Mini Kit (Qiagen, Sussex, UK) coupled with an initial bead-beating step. Briefly, 200 mg of each caecal sample were vortex-mixed in a 2 ml screw-cap tubes (Sarstedt, Wexford, Ireland) containing 0.25 g of a 1:1 mix of 0.1 mm and 1.0 mm sterile zirconia beads plus a single 3.5 mm diameter bead (BioSpec Products, Bartlesville, USA) with 1 ml of Qiagen InhibitEX® buffer. Following steps were according to manufacturer's instructions. DNA was quantified using the QubitTM 3.0 Fluorometer (Bio-Sciences, Dublin, Ireland) and the Qubit® dsDNA HS Assay Kit (Life Technologies, Oregon, USA). Extracted DNA was kept frozen at -20ºC until further analysis.

- *16S rRNA Gene Sequence-based microbiota analysis*

The V3-V4 hypervariable region of the 16S rRNA gene were amplified and prepared for sequencing as outlined in the Illumina 16S Metagenomic Sequencing Library Protocol (http://www.illumina.com/content/dam/illumina-support/documents/documentation/chemistry_documentation/16s/16s-metagenomic-library-prep-guide-15044223-b.pdf). Briefly, first PCR was done using forward primer (5'-TCGTCGGCAGCGTCAGATGTGTATAAGAGACAGCCTACGGGNGGCWGCAG-3') and reverse primer (5'-GTCTCGTGGGCTCGGAGATGTGTATAAGAGACAGGACTACHVGGGTATCTAATCC-3'). Each 25 µl PCR reaction contained 5 ng/µl microbial genomic DNA, 1 µM of each primer and 12.5 µl 2X Kapa HiFi Hotstart ReadyMix (Kapa Biosystems Ltd., UK). The PCR conditions follow as: initial denaturation at 95 ºC x 3 min; 25 cycles of 95 ºC x 30 s, 55 ºC x 30 s, 72 ºC x 30 s; and 72 ºC x 5 min for final extension. PCR products were purified with Agencourt AMPure XP system (Beckman Coulter Genomics, Takeley, UK). In the next step, dual indices and Illumina sequencing adapters were attached to PCR products using the Nextera XT Index Kit (Illumina, San Diego, CA). Each 50 µl PCR reaction contained 5 µl purified DNA, 5 µl index primer 1 (N7xx), 5 µl index primer 2 (S5xx), 25 µl 2x Kapa HiFi Hot Start Ready mix and 10 µl PCR grade water. PCR amplification was completed using the previous program but with only 8 amplification cycles instead of 25. Following this, a second clean-up step with the Agencourt AMPure XP system was done. PCR products were quantified, normalized and pooled in an equimolar fashion using the Qubit® dsDNA HS Assay Kit (Life Technologies, Oregon, USA). Next steps in the library preparation were carried out by Teagasc Next Generation DNA Sequencing Facility (Teagasc, Moorepark, Food Research Centre) prior to 2×250 (bp) paired-end sequencing on the Illumina MiSeq platform, using standard Illumina sequencing protocols.

- *Bioinformatic sequence analysis*

Bioinformatic sequence analysis was performed as previously described (1). Briefly, paired-end sequences were assembled using FLASH (2) and analysed using QIIME v1.8.0 (Quantitative Insights Into Microbial Ecology) (3). Sequences were quality checked and the remaining sequences were clustered into operational taxonomic units using USEARCH (v7-64bit) (4). Taxonomic ranks were assigned with a BLAST search against the SILVA SSURef database release 123 (5). Alpha and beta diversities and Bray-Curt dissimilarities were generated in Calypso (version 8.84) Principal coordinate analysis (PCoA) plots were visualized with ggplot2 (V 2.2.1) using OTU values normalized with the wisconson function in the vegan package (v. 2.5-1). Adonis function (PERMANOVA, permutations=999) in the vegan package (v 2.5-1) was performed on Bray-Curtis matrix on three dimensions. Relative abundance of bacterial taxa was expressed as % of identified sequences.

High performance liquid chromatography (HPLC) detection of the drugs in plasma and caecal contents

- *Plasma Sample Preparation and HPLC Conditions*

Olanzapine: The liquid-liquid extraction (LLE) protocol and HPLC conditions employed for the detection of olanzapine in plasma samples was based on a previous method with some modifications (6). 20 µl of the internal standard (I.S.), clozapine, was added to 50 µl of plasma sample (to yield final clozapine concentration of 400 ng/ml) (Discovery Fine Chemicals, UK). After sample alkalinisation with 100 µl of Na_2CO_3 (2M), 750 µl of hexane: dichloromethane: (85:15,

v/v) was added as an extraction solvent. Tubes were mixed on an eppendorf shaker at maximum speed (1400 rpm) for 5minutes at 4 °C, followed by sonication for 4 minutes (Bransonic Ultrasonic Cleaner 5510EDTH, Sigma). The clear supernatant was isolated in a new eppendorf. A further 750 µl of the extraction solvent was added to the cloudy pellet. Samples were mixed vigorously for 30 minutes and centrifuged at maximum speed for 10 minutes. The supernatant was again isolated. The tubes were dried under a stream of nitrogen (approx.15-20 minutes) to evaporate the extraction solvent and reconstituted with 100 µl of mobile phase. Compounds were eluted isocratically over a 16 min runtime at a flow rate of 1 ml/min. The mobile phase consists of 14% acetonitrile in water (containing 0.25% H_3PO_4 and 0.05% triethylamine). The limit of quantitation for risperidone was 15.6 ng/ml.

Risperidone: The liquid-liquid extraction (LLE) protocol and HPLC conditions employed for the detection of risperidone in plasma samples was based on a previous method with some modifications (7). Briefly, 100 µl of NaOH (2M) was added to 50 µl plasma samples spiked with 20 µl of I.S. (to yield final clozapine concentration of 2000 ng/ml). Tubes were vortex-mixed for 30s and 1ml diisopropyl ether-isoamyl alcohol (99:1, v/v) was added as extraction solvent. Following 10 minutes of vigorous mixing, samples are centrifuged at 4000rpm for 10 minutes. The organic phase was back extracted with 100 µl of potassium phosphate (0.1M, pH 2.2) and 30 µl of the acid solution was injected onto the HPLC column. The mobile phase consisted of acetonitrile: potassium dihydrogenphosphate [0.05M pH 3.7, pH adjusted with 25% phosphoric acid] (30:70) and was filtered through Millipore 0.22 µm Durapore filters (Millipore, Ireland). Compounds were eluted isocratically over a 12 min runtime at a flow rate of 1 ml/min. The column was maintained at room temperature and samples/standards were kept at 8 °C in the cooled autoinjector prior to analysis. The limit of quantitation for risperidone was 62.5 ng/ml.

- *Sample Preparation and Drug Extraction from Caecal Contents*

To quantify the amount of risperidone and olanzapine present in the caecum of the rats, 200mg of caecal contents from each animal was isolated and suspended in 1 ml of HPLC grade water and homogenised using a bead-beater for 3·1minute intervals. The same LLE procedure was used to process the caecal samples as that detailed for the plasma samples; the only difference involving the volume of starting material, 100 µl of the homogenised caecal content was used instead of 50 µl plasma.

- *HPLC Equipment*

The HPLC with ultraviolet detection (HPLC-UV) system consisted of Agilient 1260 Infinity Binary LC (Agilient Technologies). System components were used in conjunction with EZChrom Elite software (Mason Technology). The detector used was the 1260 Infinity II Diode Array. All samples for both drugs were injected onto a reversed phase Synergi RP 4 µm MAX-RP HPLC Column 4.60 X 250 mm column (Phenomenex), which was protected by a SecurityGuard HPLC (Phenomenex).

References

1. Murphy K, Curley D, O'Callaghan TF, O'Shea C-A, Dempsey EM, O'Toole PW, et al. The Composition of Human Milk and Infant Faecal Microbiota Over the First Three Months of Life: A Pilot Study. Scientific Reports. 2017;7:40597.
2. Magoč T, Salzberg SL. FLASH: fast length adjustment of short reads to improve genome assemblies. Bioinformatics. 2011;27(21):2957-63.
3. Caporaso JG, Kuczynski J, Stombaugh J, Bittinger K, Bushman FD, Costello EK, et al. QIIME allows analysis of high-throughput community sequencing data. Nat Methods. 2010;7.
4. Edgar RC. Search and clustering orders of magnitude faster than BLAST. Bioinformatics. 2010;26(19):2460-1.
5. Quast C, Pruesse E, Yilmaz P, Gerken J, Schweer T, Yarza P, et al. The SILVA ribosomal RNA gene database project: improved data processing and web-based tools. Nucleic Acids Research. 2013;41(D1):D590-D6.
6. Dusci LJ, Peter Hackett L, Fellows LM, Ilett KF. Determination of olanzapine in plasma by high-performance liquid chromatography using ultraviolet absorbance detection. J Chromatogr B Analyt Technol Biomed Life Sci. 2002;773(2):191-7.
7. Avenoso A, Facciola G, Salemi M, Spina E. Determination of risperidone and its major metabolite 9-hydroxyrisperidone in human plasma by reversed-phase liquid chromatography with ultraviolet detection. J Chromatogr B Biomed Sci Appl. 2000;746(2):173-81.
