# Supplementary material for: The gut microbiome influences the bioavailability of olanzapine in rats
Source: eBioMedicine. 2021 Apr 2;66:103307. doi: 10.1016/j.ebiom.2021.103307 (PMC8047500; doi:10.1016/j.ebiom.2021.103307)
Supplement: Supplementary file 3 [file mmc3.pdf]

Permission to acknowledge

I give my permission to be acknowledged in the manuscript

"The gut microbiome influences the bioavailability of olanzapine in rats" (ID EBIOM-D-19-03600R1)

(Authors: Sofia Cussotto, Jacinta Walsh, Anna V. Golubeva, Alexander V. Zhdanov, Conall R. Strain, Fiona Fouhy, Catherine Stanton, Timothy G. Dinan, Niall P. Hyland, Gerard Clarke, John F. Cryan, Brendan T. Griffin)

Signature

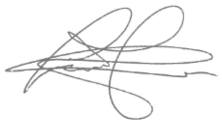

Print name

Laura Finnegan *Date* 02/03/21

Permission to acknowledge

I give my permission to be acknowledged in the manuscript

"The gut microbiome influences the bioavailability of olanzapine in rats" (ID EBIOM-D-19-03600R1)

(Authors: Sofia Cussotto, Jacinta Walsh, Anna V. Golubeva, Alexander V. Zhdanov, Conall R. Strain, Fiona Fouhy, Catherine Stanton, Timothy G. Dinan, Niall P. Hyland, Gerard Clarke, John F. Cryan, Brendan T. Griffin)

Signature

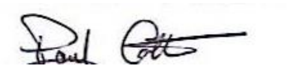

Print name

Paul Cotter *Date* 1/3/21

Permission to acknowledge

I give my permission to be acknowledged in the manuscript

"The gut microbiome influences the bioavailability of olanzapine in rats" (ID EBIOM-D-19-03600R1)

(Authors: Sofia Cussotto, Jacinta Walsh, Anna V. Golubeva, Alexander V. Zhdanov, Conall R. Strain, Fiona Fouhy, Catherine Stanton, Timothy G. Dinan, Niall P. Hyland, Gerard Clarke, John F. Cryan, Brendan T. Griffin)

Signature

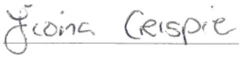

Print name

Fiona Crispie *Date* 1/3/21

Permission to acknowledge

I give my permission to be acknowledged in the manuscript

"The gut microbiome influences the bioavailability of olanzapine in rats" (ID EBIOM-D-19-03600R1)

(Authors: Sofia Cussotto, Jacinta Walsh, Anna V. Golubeva, Alexander V. Zhdanov, Conall R. Strain, Fiona Fouhy, Catherine Stanton, Timothy G. Dinan, Niall P. Hyland, Gerard Clarke, John F. Cryan, Brendan T. Griffin)

Signature

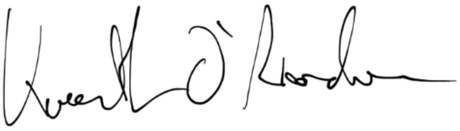

____

Print name

Kenneth O'Riordan *Date* 01 March 2021

Permission to acknowledge

I give my permission to be acknowledged in the manuscript

"The gut microbiome influences the bioavailability of olanzapine in rats" (ID EBIOM-D-19-03600R1)

(Authors: Sofia Cussotto, Jacinta Walsh, Anna V. Golubeva, Alexander V. Zhdanov, Conall R. Strain, Fiona Fouhy, Catherine Stanton, Timothy G. Dinan, Niall P. Hyland, Gerard Clarke, John F. Cryan, Brendan T. Griffin)

Signature

____

Print name

Maša Primožič *Date* 25.2.2021

Permission to acknowledge

I give my permission to be acknowledged in the manuscript

"The gut microbiome influences the bioavailability of olanzapine in rats" (ID EBIOM-D-19-03600R1)

(Authors: Sofia Cussotto, Jacinta Walsh, Anna V. Golubeva, Alexander V. Zhdanov, Conall R. Strain, Fiona Fouhy, Catherine Stanton, Timothy G. Dinan, Niall P. Hyland, Gerard Clarke, John F. Cryan, Brendan T. Griffin)

Signature

____

Print name

Patrick Fitzgerald

Date 25th February 2021
